# Stromal CCL2/CXCL10/SPARC axes drive pro-tumor CHI3L1 expression for microenvironmental modulation in pancreatic cancer progression and therapeutic innovation

**DOI:** 10.1186/s12929-026-01295-2

**Published:** 2026-10-26

**Authors:** Pei-Chia Su, Yan-Shen Shan, Ya-Chin Hou, Po-Hsien Huang, Pao-Chi Liao, Yi-Ching Wang

**Affiliations:** 1 Department of Pharmacology, College of Medicine, National Cheng Kung University, No. 1, Ta-Hsueh Road, Tainan, 70101 Taiwan; 2 Department of Clinical Medical Research, College of Medicine, National Cheng Kung University, Tainan, Taiwan; 3 Institute of Clinical Medicine, College of Medicine, National Cheng Kung University, Tainan, Taiwan; 4 Department of Biochemistry and Molecular Biology, College of Medicine, National Cheng Kung University, Tainan, Taiwan; 5 Department of Environmental and Occupational Health, College of Medicine, National Cheng Kung University, Tainan, Taiwan; 6 Department of Basic Medical Sciences, College of Medicine, National Cheng Kung University, Tainan, Taiwan

**Keywords:** Chitinase-3-like-protein 1, Pancreatic cancer, Cancer-stromal cells co-culture, Transcriptional regulation, Epigenetic reprogramming

## Abstract

**Background:**

Chitinase-3-like-protein 1 (CHI3L1), a low-abundance protein in normal tissues, is markedly upregulated in the pancreatic tumor microenvironment (TME), where it promotes fibrosis and immune evasion. Spatial transcriptomics revealed enrichment of CHI3L1 at tumor-stroma interfaces, suggesting that cell-cell interactions regulate CHI3L1 expression.

**Methods:**

We employed 2D and 3D multicellular culture models, incorporating pancreatic cancer cells, macrophages, and pancreatic stellate cells (PSCs), to recapitulate TME-mediated CHI3L1 induction. Mechanistic insights were gained through secretome profiling, chromatin immunoprecipitation (ChIP), and methylation-specific PCR. The therapeutic potential of targeting identified axes was evaluated in an orthotopic pancreatic adenocarcinoma (PDAC) mouse model.

**Results:**

We identified CCL2, CXCL10, and SPARC as key secreted mediators during tumor-stroma crosstalk that cooperatively induced CHI3L1 expression in cancer cells via activation of NF-κB and STAT3. Specifically, inflammatory cytokines within the TME recruited macrophages to induce CCL2, which, in concert with PSCs, enhances CXCL10 and SPARC secretion. Mechanistically, NF-κB directly bound the *CHI3L1* promoter, while STAT3 reduced DNA methyltransferase 1 levels, leading to promoter hypomethylation and epigenetic activation of CHI3L1. Clinically, high CHI3L1 expression correlated with the upregulated stromal CCL2, CXCL10, and SPARC levels, and the combined four-marker signature was an independent prognostic factor for both overall and disease-free survival in multivariable analysis. In vivo, blockade of the upstream stromal pathways (CCR2/CXCR3/TGFβR1) coupled with CHI3L1 neutralization reduced tumor growth and remodeled the immunosuppressive microenvironment in immunocompetent PDAC models.

**Conclusions:**

Our findings uncover a cooperative multicellular signaling network that orchestrates CHI3L1 expression in cancer via transcriptional and epigenetic reprogramming. Disrupting these upstream stromal pathways concurrently with CHI3L1 neutralization was associated with reduced T-cell exhaustion, offering a promising combinatorial approach for PDAC immunotherapy.

**Supplementary Information:**

The online version contains supplementary material available at https://doi.org/10.1186/s12929-026-01295-2.

## Background

Pancreatic ductal adenocarcinoma (PDAC) stands out as one of the most aggressive forms of cancer, boasting an alarmingly low five-year relative survival rate. Predictions indicate that PDAC will rise to become the second leading cause of cancer-related fatalities by the year 2030 [1]. Currently, the first-line therapeutic methods of pancreatic cancer, FOLFIRINOX and gemcitabine combined with nanoparticle albumin-bound paclitaxel, could only slightly improve median overall survival [2].

The tumor microenvironment (TME) is constituted of cancer cells and stromal cells, including diverse immune cells, cancer-associated fibroblasts (CAF), endothelial cells, and other tissue-resident cells [3]. Increasing evidence indicates that various stromal cells play distinct roles in tumor development, progression, and drug resistance through diverse mechanisms [4]. PDAC is distinguishable by an abundance of desmoplasia, and pancreatic stellate cells (PSCs) are one of the most important stromal fibroblasts in this stroma-rich carcinoma [5, 6]. This dense extracellular matrix not only acts as a physical barrier but also hampers immune cell infiltration and antitumor defenses. Orthotopic models by injecting the mixture of PSCs and pancreatic cancer cells accelerate tumor growth and metastasis [7]. Collectively, cross-talks between stromal cells and cancer cells are crucial drivers of cancer progression and metastasis.

Chitinase-3-like-protein 1 (CHI3L1) has been found to be important in regulating TME. CHI3L1 is a secreted protein produced by a variety of cells within TME, including macrophages, T cells, fibroblasts, neutrophils, and tumor cells [8–11]. The significant increase in CHI3L1 levels triggers the polarization of M2 macrophages by activating the interleukin-13 receptor alpha-2 (IL-13Rα2)-MAPK signaling pathway [12, 13]. Elevated CHI3L1 within T cells is prone to differentiate into the Th2 phenotype, and secreted CHI3L1 has been proven to increase the ratio of regulatory T cells (Treg) while reducing CD8^+^ T cell cytotoxicity [14, 15]. Beyond its role in immune regulation, CHI3L1 has been implicated in promoting epithelial-to-mesenchymal transition in gastric cancer by activating the CD44/β-catenin/Erk/Akt signaling pathway [16]. Moreover, CHI3L1 participates in extracellular matrix remodeling [17]. Studies have revealed the angiogenesis-supporting effect of CHI3L1 through directly promoting tube formation of endothelial cells and indirectly recruiting M2 macrophages to produce vascular endothelial growth factor [10]. Increasing evidence supports the relationship between CHI3L1 and tissue fibrosis [18].

Elevated CHI3L1 in serum and intra-tumoral expression correlates with tumor metastasis and poor outcome of cancers [19]. Nonetheless, most epithelial cells expressed low levels of CHI3L1, which were largely increased during cancer progression [20]. Notably, PDAC is characterized as an immune-cold tumor with severe dysplasia; most CHI3L1-expressing cells are barely detectable in the pancreatic TME. Therefore, dissecting the mechanism by which cancer cells acquire CHI3L1 expression for their progression is an urgent issue.

The transcriptional regulation and signal regulation of CHI3L1 have been reported. For example, transcription factor (TF) SP-1 markedly increased CHI3L1 expression during the late stages of macrophage differentiation [21]. Notably, pro-inflammatory cytokines such as IL-1 and IL-6 induce CHI3L1 expression via STAT3 and NF-κB p50 complexes in astrocytes during sterile inflammation [22, 23]. The first report for signal regulation of CHI3L1 in cancer cells was published in 2005, which showed that hypoxia condition in cancer increased the expression of CHI3L1, while TNFα and basic fibroblast growth factor downregulated CHI3L1 expression in glioblastoma (GBM) cell lines [24]. GBM cell lines constitutively expressed a high basal level of CHI3L1 compared to other cancer cell lines, reflecting the highly activated PI3K-AKT pathway in GBM cells [13].

In this study, we delineate how interactions between pancreatic cancer cells and stromal components of the TME drive CHI3L1 upregulation in PDAC. Using 2D and 3D mixed-culture systems that recapitulate the PDAC TME, we show that crosstalk among cancer cells, macrophages, and PSCs robustly induces CHI3L1 expression. Mechanistically, macrophage-derived CCL2 promotes SPARC secretion from PSCs, while CXCL10 emerges only under multicellular interactions. Together, these soluble factors synergistically induce CHI3L1 through NF-κB-dependent transcriptional activation and STAT3-mediated promoter demethylation. Importantly, combinatorial targeting of these upstream receptor axes together with CHI3L1 neutralization not only restricts tumor burden and fibrosis but also restores anti-tumor cytotoxic immunity in immunocompetent models, demonstrating a viable strategy to reprogram the PDAC immune microenvironment.

## Materials and methods

### Cell culture

Human pancreatic cancer cell lines MiaPaCa-2, PANC-1, and BxPC-3, human Jurkat T cells, human monocytes THP-1, human pancreatic stellate cells (PSC), and human HEK293T cells were purchased from the American Type Culture Collection. Murine KPPC cancer cells were isolated and established from primary pancreatic tumors of spontaneous *LSL-Kras*^*G12D/*+^*; Trp53*^*fl/fl*^*; Pdx1-Cre* KPPC mice (KPPC) transgenic mice [27] and stably transduced with a lentiviral vector encoding firefly luciferase (KPPC-luc) for in vivo bioluminescence monitoring. MiaPaCa-2, PANC-1, HCT116, Jurkat T, and THP-1 were cultured in RPMI (Gibco, Grand Island, NY, USA); BxPC-3, PSCs, H1299, KYSE510, and HEK293T were cultured in Dulbecco’s modified Eagle’s medium (DMEM, Gibco) at 37 °C in 5% CO_2_ humidified atmosphere. All media were supplemented with 10% fetal bovine serum (FBS, Corning, NY, USA) and 1% penicillin/streptomycin (PS, GeneDireX, Taoyuan, Taiwan). The medium for BxPC-3 was further supplemented with 1% non-essential amino acids (NEAA, Gibco) and 1% sodium pyruvate (Gibco). Primary human peripheral blood mononuclear cells (hPBMCs) were cultured in RPMI medium containing 10% FBS, 1% PS, and 20 *n*g/ml human M-CSF (Peprotech, Cranbury, NJ, USA). Serum-free RPMI medium was supplemented with 1% PS, 1% NEAA (Gibco), and 1.5 mM HEPES (Gibco).

### Cancer-stromal cells culture medium preparation and proteomic secretome profiling

Firstly, THP-1 cells were seeded in a 10 cm dish and differentiated into macrophages with phorbol 12-myristate 13-acetate (PMA, Abcam, Cambridge, UK) at 20 *n*g/ml for 48 h. Cancer cells and PSC cells were then directly mixed with or without macrophages and cultured for 7 h. Each cell type was seeded at a concentration of 3 × 10^6^ for monoculture, 1.5 × 10^6^ for double-culture, and 1 × 10^6^ for triple culture, and 5 × 10^5^ Jurkat T cells were added only to the CSMT condition. Subsequently, culture media were removed and washed with 1 × PBS three times, then cultured in serum-free media for 40 h. The supernatants of cancer cells (C), PSCs (S), and macrophages (M) double (CS or CM) or triple (CSM) cultures were centrifuged at 1,400 rpm for five min to remove cell debris and filtered through a 0.22 µm filter. These culture media were named C, CS, CM, or CSM, respectively. For all conditioned-medium education experiments, cell numbers and culture duration were held constant across conditions (see above), and conditioned media were applied at a fixed volume rather than normalized to total protein concentration, to preserve the CSM-specific increase in secreted factors (e.g., CCL2, CXCL10, SPARC) as the biological signal of interest.

For proteomic secretome profiling, the culture media were concentrated by centrifugation through a 3 kDa molecular weight cutoff Ultracel® membrane (Millipore, Darmstadt, Germany) at 4,000*g* for two hours at 4 °C. Protein concentration was measured in the concentrated culture media, and 100 µg of each mixed-culture media was aliquoted. Three biological replications of culture media were collected for mass spectrometry.

### Generation and culture of human PDAC spheroids

Spheroids were developed using a 24-well plate (Acrocyte, Taiwan) [25]. A total of 1 ml of PBS containing 5% PS was used for plate recovery for 15 min before seeding cells. After recovery, the plate was washed with 1× PBS twice and replenished with 1 ml of the Initial medium (Acrocyte). To generate spheroids, 1 × 10^4^ BxPC-3 were prepared for monoculture; A total of 1 × 10^4^ cells, including BxPC-3, PSC, and primary human macrophages in the ratio of 1:1:1 for triple culture, were suspended in 100 µl of “Initial medium” and incubated at 37 °C for 20 min to increase cell-cell contact before seeding into the medium-containing well. The size and integrity of the spheroids were monitored for 14 days through image analysis. The media were replenished with 500 µl of “Maintaining medium” (Acrocyte) every three days. The spheroids were collected at the end of day 14. After centrifuging at 700 rpm for 5 min, spheroids were washed with 1× PBS and resuspended in O.C.T for cryosection and further validation.

### Cell-surface co-immunoprecipitation (co-IP)

MiaPaCa-2 and PANC-1 cells were incubated with recombinant SPARC (1 μg/ml) for 1 h at 4 °C to allow cell-surface binding while preventing receptor internalization. Cell-surface protein complexes were then stabilized by chemical cross-linking with the membrane-impermeable cross-linker bis(sulfosuccinimidyl)suberate (BS3, 2.5 mM, 30 min, 4 °C, MedChemExpress, Monmouth Junction, NJ, USA), followed by quenching with 25 mM Tris-HCl (pH 7.5) for 15 min at 4 °C. Cells were lysed in IP buffer (50 mM Tris, 300 mM NaCl, 2 mM EDTA, 2% NP-40, 10% glycerol, pH 7.4) containing protease inhibitor cocktail, and TGFβR1 was immunoprecipitated from the lysate using an anti-TGFβR1 antibody or normal IgG as a negative control. Co-immunoprecipitated SPARC and TGFβR1 were detected by immunoblotting; GAPDH served as a loading control for input lysates. The antibodies used and the experimental conditions are listed in Table S1.

### Chromatin immunoprecipitation assay (ChIP assay)

ChIP was performed on MiaPaCa-2 or PANC-1 cells. Cell lysates were cross-linked with 1% formaldehyde, followed by preparation of nuclear lysate using the Magna ChIP™ protein G kit (Millipore). Nuclear lysates were sonicated to shear DNA to around 500 bp using a QSONICA sonicator with cooling system (on 45 s, off 30 s, 40 cycles) followed by immunoprecipitation for 16 h at 4 °C using 1 µg anti-phospho-NF-κB antibody (Cell Signaling, Danvers, MA, USA), anti-phospho-STAT3 antibody (Cell Signaling), anti-DNMT1 (GeneTex) or normal rabbit IgG (negative control, Novus, Littleton, CO, USA). The levels of targeted genes in ChIP products were determined by quantitative reverse transcription PCR (RT-qPCR). The total input served as internal control. Primers are listed in Table S2.

### Cycloheximide (CHX) chase assay

MiaPaCa-2 were transfected with a STAT3-expressing plasmid or the corresponding empty vector for 24 h. Cells were then treated with cycloheximide (20 µg/ml, Sigma-Aldrich) to block de novo protein synthesis and harvested at 0, 6, 12, and 24 h after CHX addition. Cell lysates were prepared, and DNMT1 protein levels were determined by immunoblotting as described above, with GAPDH as a loading control. Band intensities were quantified using ImageJ, normalized to the loading control, and expressed as the percentage of the level at time 0 to estimate the relative decay rate of DNMT1. Each experiment was performed in three biological replicates.

### Promoter constructs and dual luciferase promoter assay

Human *CHI3L1* promoter region (− 2133 to − 145 of the transcriptional start site, TSS) was inserted into the *Kpn*I and *Xho*I sites of pGL4.17 luciferase expression vector, yielding pGL4.17-hCHI3L1-NF-κB-1, pGL4.17-hCHI3L1-NF-κB-2, pGL4.17-hCHI3L1-NF-κB-3, and pGL4.17-hCHI3L1-NF-κB-4. Primers for generating CHI3L1 promoter containing one to four NF-κB binding sites were listed in Table S2. Site-directed mutagenesis was used to generate pGL4.17-hCHI3L1-NF-κB, in which the consensus binding sequence of NF-κB was disrupted using Novagen KOD Polymerase PCR system (Millipore). To confirm if mixed-cultured media regulated CHI3L1 through transcription, the luciferase reporter plasmids pGL4.17-hCHI3L1-1 to pGL4.17-hCHI3L1-NF-κB-4 were co-transfected with pGL4-Renilla plasmid in a 10:1 ratio into MiaPaCa-2 cells with C or CSM mixed-culture media education for 24 h. To confirm which NF-κB binding site is required for mixed-cultured media to induce CHI3L1 transcription, the luciferase reporter plasmids pGL4.17-hCHI3L1-NF-κB-4 and pEGFP-NF-κB-p65 were co-transfected in a 1:1 ratio into MiaPaCa-2 cells. Luciferase activity was determined using the Dual-Glo Luciferase assay system (Promega, Madison, USA). After 24 h of plasmid transfection, cells were lysed in 1× passive lysis buffer (Promega). A 10 µl aliquot of cell lysate was mixed with 10 µl of Dual-Glo Luciferase substrate for CHI3L1 promoter-driven luciferase activity determination. Luminescence was detected using SpectraMax iD3.

### Bisulfite conversion, methylation-specific PCR (MSP), and pyrosequencing

Genomic DNA (gDNA) of MiaPaCa-2 was purified using the Quick-DNATM Microprep Plus kit (Zymo Research, California, USA). Next, the gDNA was treated with sodium bisulfite to convert unmethylated cytosine to uracil, using the EZ DNA Methylation-GoldTM kit (Zymo Research). For MSP analysis, the promoter methylation status of CHI3L1 was assessed using EpiTaq™ HS (for bisulfite-treated DNA; TaKaRa) with specific methylated (M) and unmethylated (U) primer pairs. Amplification was conducted for 30 cycles at annealing temperatures of 49 °C (U reaction) or 50 °C (M reaction). Primer sequences are listed in Table S2. To quantitatively evaluate site-specific methylation levels, bisulfite-converted DNA was amplified by PCR using biotinylated primers targeting the *CHI3L1* promoter. Biotinylated amplicons were immobilized onto Streptavidin Sepharose High Performance beads (Cytiva, Marlborough, MA, USA), denatured with 0.2 M NaOH, and processed to single strands using the PyroMark Q96 Vacuum Workstation (QIAGEN, Hilden, Germany). Following sequencing primer annealing at 80 °C for 2 min, quantitative pyrosequencing was conducted on a PyroMark Q96 ID platform (QIAGEN) using PyroMark Gold Q96 reagents. Methylation percentages across individual CpG sites were calculated using PyroMark CpG software (QIAGEN).

### Plasmid transfection, lentivirus preparation, and infection

Plasmids used in the study are listed in Table S3. For PDAC cell lines, the plasmid was transduced using Lipofectamine 2000 reagent (Thermo Fisher Scientific, Waltham, MA, USA) according to the manufacturer’s protocol. The lentiviral shRNA-expressing plasmid for human RelA, STAT3, CCL2, CXCL10, SPARC, CXCR3, CCR2, and TGFβR1, pLKO_TRC001, and packaging plasmids (containing pMD.G and pCMVΔ8.91) were purchased from RNA Technology and Gene Manipulation Core (Taipei, Taiwan). The lentiviral shRNA-expressing plasmids were co-transfected with packaging plasmids according to the manufacturer's instructions. After 72 h of transfection, the lentiviral-containing media were collected and aliquoted. MiaPaCa-2 and PSC were infected with lentivirus using 8 μg/ml polybrene, PANC-1 was infected using 6 μg/ml polybrene, and THP-1 was infected using 2 μg/ml polybrene. The infected cells were selected by treatment with 2 µg/ml puromycin.

### Tissue collection and spatial whole-transcriptome atlas

Tumor specimens were obtained from three PDAC patients undergoing surgical resection at the National Cheng Kung University Hospital (NCKUH) under an Institutional Review Board (IRB)-approved protocol (IRB number: NCKUH B-ER-110–420) [26]. Formalin-fixed, paraffin-embedded (FFPE) PDAC tissue samples were processed using the GeoMx Digital Spatial Profiling (DSP) RNA assay with the Whole Transcriptome Atlas (NanoString), following the manufacturer’s standard protocol. Morphological profiling was achieved using DSP barcodes conjugated to UV-photocleavable oligonucleotides linked to antibodies against PanCK (epithelial cell marker), CD45 (immune cell marker), and SYTO13 (nuclear stain). High-resolution whole-slide scanning was performed using a ×20 objective lens on the DSP instrument to define and select regions of interest (ROIs). UV light was then applied to cleave the oligos from each ROI, which were subsequently collected, PCR-amplified, and sequenced using the Illumina NovaSeq platform.

### Single-cell RNA sequencing and data processing

Raw scRNA-seq FASTQ files from two 10× Genomics studies were processed using CellRanger (v6.1.1) and aligned to the GRCh38. Downstream analysis used Seurat (v4.3.1). Cells (< 100 genes, > 50% mitochondrial genes, < 500 transcripts) were excluded, leaving 34,437 cells, which were merged, normalized, and scaled with adjustments for feature number, UMI counts, and mitochondrial percentage. After principal component analysis (PCA), cells were clustered with the Leiden algorithm and visualized with UMAP. Major cell types were annotated, and differentially expressed genes were identified by the Wilcoxon rank-sum test (FDR < 0.05).

Cell–cell communication analysis was inferred separately for ADJ, PT, and MET using CellChat (v1.6.1) with the full CellChatDB.human database. Communication probabilities were computed with computeCommunProb (raw.use = TRUE, trim = 0.1). Interactions with fewer than 3 cells per group (filterCommunication, min.cells = 3) were excluded, and significance was assessed by permutation testing (100 permutations, *P* < 0.05). Pathway-level and aggregate networks were visualized as circle plots, with edge widths normalized to a common maximum across groups and edge colors indicating signaling sources.

### Spatial transcriptomics and trajectory analysis

Gene expression profiling for spatial transcriptomics was conducted using the Visium Spatial platform by 10× Genomics (dataset GSE235315) and analyzed using Seurat. Spots expressing fewer than 500 genes or more than 8,000 genes or with a mitochondrial ratio exceeding 10% were excluded. Data was normalized using the LogVMR method and dimensionality reduction and clustering was performed by PCA, with the first 30 principal components (PCs) and a clustering resolution of 2. Spatial feature expression plots were generated using Seurat (v4.3.1).

Spatial trajectories were reconstructed using Monocle3 (v1.3.7) in R. A cell_data_set object was constructed from the normalized spatial gene expression matrix using the spatial cluster assignments and UMAP coordinates from the Seurat analysis above. After preprocessing and clustering (cluster_cells), the trajectory graph was learned (learn_graph, use_partition = FALSE), and pseudotime was calculated by ordering spots along the graph (order_cells), with the root defined as the tumor-only cluster. Because 10× Visium spots (55 μm diameter) capture multicellular mixtures rather than individual cells, spatial pseudotime values reflect a continuum of transcriptional similarity across tissue microenvironments and gene expression gradients, rather than confirmed single-cell lineage differentiation or a validated temporal sequence.

### PDAC orthotopic model

All mice (6 to 8-week-old male mice) were maintained under standard laboratory conditions (temperature 23 ± 1 °C, humidity 40-60%, 12-h light/dark cycle). The mice were acclimatized for two weeks. The Institutional Animal Care and Use Committee (IACUC Approval No. 110332) of the National Cheng Kung University reviewed and approved all protocols. For orthotopic implantation, MiaPaCa-2 cells were injected into the pancreas of NOD-SCID mice, and syngeneic KPPC-luc cells were injected into the pancreas of C57BL/6J mice. Mice were randomized into treatment cohorts (n = 7 per group). Vehicle control; CCR2 inhibitor monotherapy (RS 504393, 5 mg/kg, i.p., every other day; MedChemExpress); CXCR3 inhibitor monotherapy (AMG 487, 5 mg/kg, i.p., every other day; MedChemExpress); TGFβR1 inhibitor monotherapy (Vactosertib, 10 mg/kg, o.g., daily; MedChemExpress); a triple-inhibitor regimen (RS 504393, AMG 487, and Vactosertib combined at the above doses and schedules), and a combination regimen (triple-inhibitor regimen plus CHI3L1 nAb, the anti-CHI3L1 neutralizing antibody characterized by our previous study [11] (10 mg/kg, i.v., once weekly; Leadgene Biomedical, Inc, Taiwan). RS 504393, AMG 487, and Vactosertib were each formulated in a vehicle comprising 10% DMSO, 40% PEG300, 5% Tween-80, and 45% saline. Vehicle-treated control (ctrl) mice received the same vehicle formulation as the triple-inhibitor regimen (i.p. every other day; o.g. daily), together with normal mouse IgG (10 mg/kg, i.v., once weekly) in place of CHI3L1 nAb, so that the control group received an equivalent volume, route, and frequency of vehicle/isotype administration as the combination group. Tumor burden was monitored by IVIS bioluminescence imaging. Upon sacrifice, tumors, lymph nodes, peripheral blood, and vital organs were collected for histological, flow cytometric, and toxicity assessments.

### Multiplex immunofluorescent (IF)-IHC staining

IF staining was conducted to detect CHI3L1, pan-cytokeratin (panCK), Cytokeratin 19 (CK19), CXCL10, CCL2, SPARC, CD68, ACTA2, CD86, CD206, CD8, PD-1, LAG3, and Granzyme B expression within TMA of PDAC patients, tumor sections from KPPC mice (PMID: 32552862) [27], PDAC-orthotopic mice tumor sections, or 3D spheroids using the Opal 4-color Manual IHC Kit (AKOYA Biosciences, Marlborough, MA, USA) according to the manufacturer's instructions. The IF staining protocol is consistent with the IHC protocol before blocking. The IF slides were blocked with antibody diluent (Dako, Santa Clara, CA, USA) for 10 min at room temperature. Following, the slides were washed with Tris-Buffered Saline with Tween® 20 (TBST; Sigma) once and conjugated with anti-CHI3L1, panCK, CK19, CXCL10, CCL2, SPARC, CD68, ACTA2, CD86, CD206, CD8, PD-1, LAG3, or Granzyme B antibodies at 4 °C overnight. The slides were then washed with TBST three times and incubated with Opal Polymer HRP mouse + rabbit reagent for 10 min at room temperature. After washing with TBST three times, the Opal fluorophore (1:200 dilutions with 1×Plus amplification diluent) was added and incubated for 10 min at room temperature. The fluorescent antibody reaction and the subsequent steps were performed in the dark. The slides were washed three times with TBST. Lastly, the DAPI mounting solution (Fluoroshield with DAPI, GeneTex) was applied for nucleus staining and mounting. The quantification was performed using a semi-quantitative scoring system based on immunofluorescence intensity and the percentage of positive cells. The staining intensity was categorized on a scale of 1 to 5. For the PDAC allograft, measurements were performed on more than six non-overlapping ROI using ImageJ software. For survival analysis, staining intensity for CHI3L1, CCL2, CXCL10, and SPARC was graded relative to the intensity distribution across all patients in the cohort, into three categories: High (upper intensity tertile), Medium (intermediate intensity), and Low (lower intensity/weak immunoreactivity). Patients were classified as "four-high" if all four markers were scored as High; all other patients were classified as "Others". The antibodies used and the experimental conditions are listed in Table S1.

### Patient samples and clinical information

Tumor sections from surgically resected PDAC patients (56 and 57, for IHC and IF-IHC, respectively) and pancreatic juice from 43 patients were recruited from NCKU Hospital after obtaining appropriate institutional review board approval (#B-ER-106-102) and informed consent from the patients. Overall survival and disease-free survival were calculated from the day of surgery to the date of death, date of cancer recurrence, or the last follow-up. The end of the follow-up was defined as June 2018.

### Statistical analysis

Unless otherwise stated, in vitro experiments (immunoblotting, luciferase reporter assays, ChIP-qPCR, and RT-qPCR) were performed with a minimum of three independent biological replicates, and representative results are shown. The data were presented as Mean ± SD and analyzed using GraphPad Prism software 8.0. Student’s t-test was employed for comparing two groups, while one-way analysis of variance (ANOVA) was used for comparing multiple groups. Differences in normalized CHI3L1 expression across spatial clusters were assessed using the Kruskal–Wallis test for overall comparison among the five clusters, followed by pairwise Wilcoxon rank-sum tests with Benjamini-Hochberg (BH) correction for multiple comparisons. Statistical analyses of the level of intra-tumoral protein expression and patients’ survival time were performed using the Statistical Package for the Social Sciences (SPSS) version 26.0 (SPSS Inc., Headquarters, Chicago, IL, United States). Overall survival (OS) and disease-free survival (DFS) curves were calculated using the Kaplan–Meier method and assessed by the log-rank test. For the CHI3L1/CCL2/CXCL10/SPARC four-high signature, univariate and multivariable Cox proportional hazards regression analyses were additionally performed to estimate hazard ratios (HRs) and 95% confidence intervals (CIs) for OS and DFS. The multivariable model adjusted for age, T stage, and N stage. Statistical significance was defined as a *P*-value < 0.05.

### Immunoblotting, RNA extraction and quantitative reverse transcription PCR (RT-qPCR), enzyme-linked immunosorbent assay (ELISA), pseudotime trajectory of single-cell analysis, flow cytometry, immunochemistry (IHC) assay, and picro-sirius red (PSR) staining assay

The detailed protocols were described in Supplementary Materials and Methods.

## Results

### Crosstalk among cancer cells, fibroblasts, and macrophages promotes CHI3L1 induction in cancer cells during pancreatic tumor progression.

**Fig. 1:**
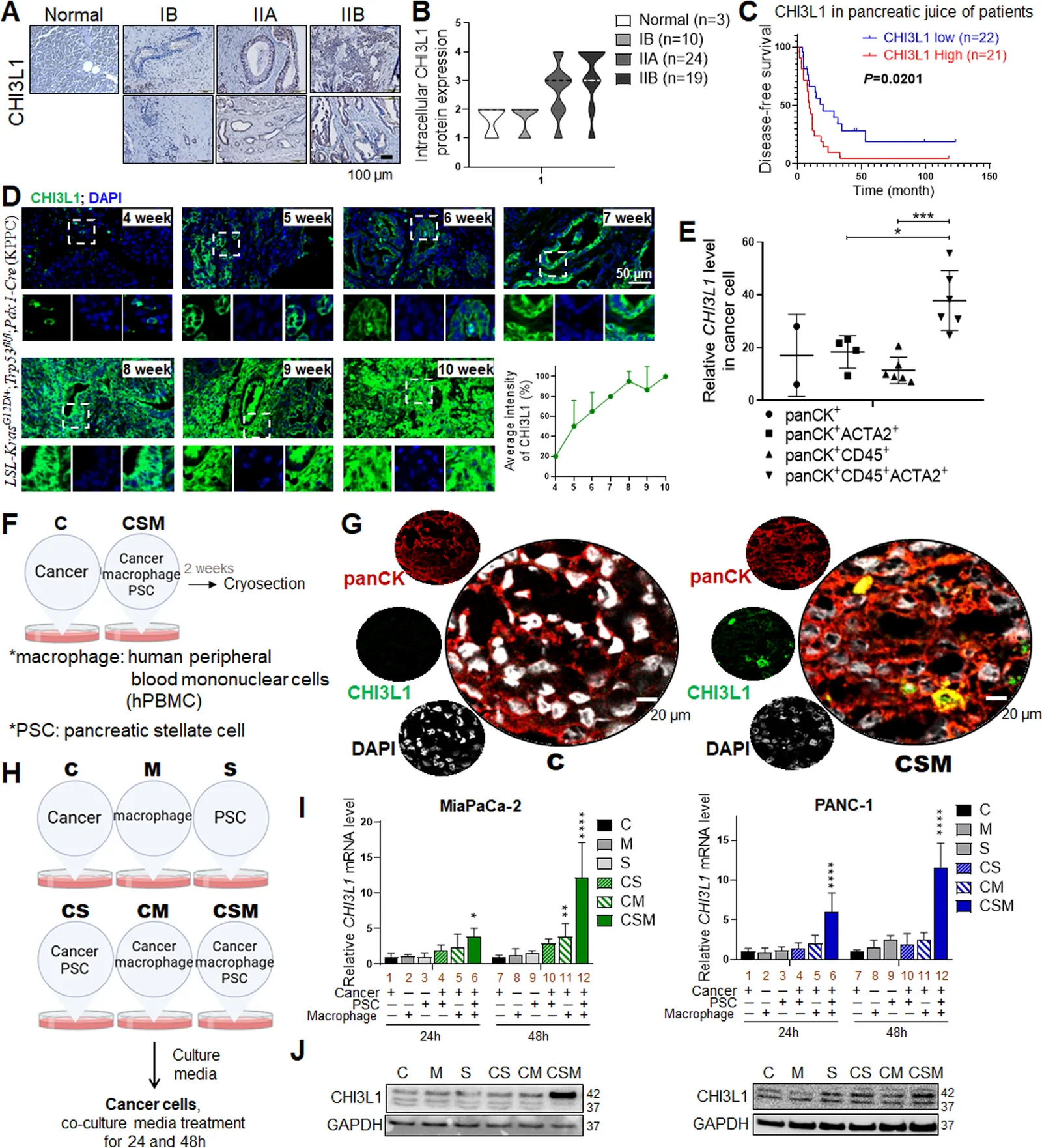
CHI3L1 expression in cancer cells is primarily induced by cancer-stromal cell interaction. **A** Representative images of IHC-stained slides of PDAC patients at different stages, showing the gradual increase in the expression of CHI3L1 (Scale bar, 100 µm). **B** Statistical analysis of IHC-stained slides. n = 3 for normal; n = 10 for stage IB; n = 24 for stage IIA; n = 19 for stage IIB. **C** Correlation between disease-free survival and secreted CHI3L1 in the pancreatic juice of PDAC patients. (n = 21 and 22 for each group). **D** Representative images of IF-IHC-stained slides from *LSL-Kras*^*G12D*/+^; *Trp53*^*fl*/*fl*^; *Pdx1-Cre* (KPPC) spontaneous PDAC mouse model showing the expression of CHI3L1 (green), scale bar, 50 µm. Statistical analysis of mouse IHC-stained slides (lower right). **E** The transcript reads of *CHI3L1* within cancer cells located in different regions of interest (ROI). PanCK^+^: cancer epithelial cells, ACTA2^+^: pancreatic stellate cells, CD45^+^: immune cells. **F** Model of cancer cells (C), pancreatic stellate cells (PSC, S), and primary macrophages (M) from human peripheral blood mononuclear cells used as a CSM triple-culture in 3D spheroids. **G** CHI3L1 protein expression (green), cancer (panCK, red), and nucleus (DAPI) in 3D spheroids under mono- (C) or triple-culture (CSM) conditions. (Scale bar, 20 µm). **H** Experimental model of an in vitro 2D tumor microenvironment-mimetic system. **I** *CHI3L1* mRNA levels in MiaPaCa-2 and PANC-1 cultured with media collected from mixed cultures of cancer cells (C), PSC (S), macrophages (M), as well as double cultures of CS, CM, and/or CSM triple-culture media. **J** CHI3L1 protein levels after 48 h of treatment with different mixed culture media. Data are presented as mean ± SD. *P* values were determined by one-way ANOVA (*P < 0.05, **P < 0.01, ***P < 0.001, ****P < 0.0001)

CHI3L1 expression is regulated by context-dependent transcriptional programs [22–24]. In our in-house cohort, normal pancreatic epithelium exhibited minimal CHI3L1 levels, whereas PDAC patient samples showed strong CHI3L1 expression localized predominantly to tumor epithelial cells, with limited stroma staining (Fig. 1A, B, Fig. S1A). Data from The Human Protein Atlas confirmed significantly higher CHI3L1 protein levels in PDAC tissues relative to normal pancreas (Fig. S1B). Additionally, Higher CHI3L1 levels in pancreatic juice were also associated with reduced disease-free survival, underscoring its clinical relevance (Fig. 1C).

These findings were validated *in vivo* using the *LSL-Kras*^*G12D*/+^; *Trp53*^*fl*/*fl*^; *Pdx1-Cre* (KPPC) spontaneous PDAC model [27], where intra-tumoral CHI3L1 expression progressively increased with disease progression (Fig. 1D). In contrast, cytokines previously reported to induce CHI3L1 in other contexts (IL-4, IL-6, IL-13, IFNγ) failed to induce CHI3L1 in PDAC cell lines, suggesting alternative regulatory mechanisms (Fig. S1C).

Spatial transcriptomic analysis revealed that CHI3L1 expression in cancer cells was enriched in regions adjacent to fibroblasts and immune cells (panCK⁺ACTA2⁺CD45⁺), compared with cancer-only regions (Fig. 1E, Fig. S1D). Correspondingly, CHI3L1 protein was preferentially detected in areas enriched with macrophages (F4/80⁺) and fibroblasts (αSMA⁺) (Fig. S1E), indicating that stroma and immune interactions within the TME may promote CHI3L1 expression in PDAC.

To mechanistically assess TME-driven regulation, we utilized a 3D spheroid system incorporating key stroma and immune components. Pancreatic cancer cells (C) were cultured alone or together with PSCs (S) and macrophages (M), representing the major PDAC TME cell types (Fig. 1F). Immunofluorescence (IF) confirmed the presence and spatial organization of all three cell populations (Fig. S1F). Notably, CHI3L1 expression in cancer cells was elevated under triple culture (CSM) conditions compared with monoculture (C), indicating that TME-derived signals drive CHI3L1 induction (Fig. 1G).

To assess whether CHI3L1 induction in cancer cells is mediated by direct contact or soluble TME-derived factors, we performed conditioned-media experiments. Cancer cell lines (MiaPaCa-2 and PANC-1) were treated with media collected from monoculture (C), dual cultures, or triple cultures (CSM) (Fig. 1H). *CHI3L1* mRNA levels were most significantly and time-dependently increased in cells exposed to CSM media (Fig. 1I), accompanied by elevated CHI3L1 protein (Fig. 1J). Together, these results support that coordinated cancer-stroma-macrophage interactions are required to induce CHI3L1 expression by cancer cells.

Considering that PDAC contains limited T-cell infiltration, we examined a four-cell culture incorporating T cells (CSMT). *CHI3L1* mRNA and protein levels were comparable between CSM and CSMT conditions (Fig. S1G, H), indicating that T cells did not significantly contribute to CHI3L1 induction. Collectively, these results demonstrate that paracrine signals generated through cancer-stroma-macrophage interactions are the primary driver of CHI3L1 upregulation in cancer cells.

### Cancer-stroma interaction releases CCL2, CXCL10, and SPARC for CHI3L1 upregulation

To identify factors in mixed-culture media that induce CHI3L1 in pancreatic cancer cells, we first heat-denatured the media (100 °C for 10 min) (Fig. S2A), which abolished CHI3L1 induction, indicating a requirement for heat-labile protein components (Fig. S2B). We then performed secretome profiling using serum-free conditioned media from monoculture (C), dual cultures (CS and CM), and triple cultures (CSM). Minimal cell lysis (< 1%) was confirmed by Actin and GAPDH controls (Fig. S2C), validating sample quality. Unsupervised hierarchical clustering revealed distinct secretion profiles, with proteins enriched in CS and CM also consistently elevated in CSM (Fig. S2D).

**Fig. 2:**
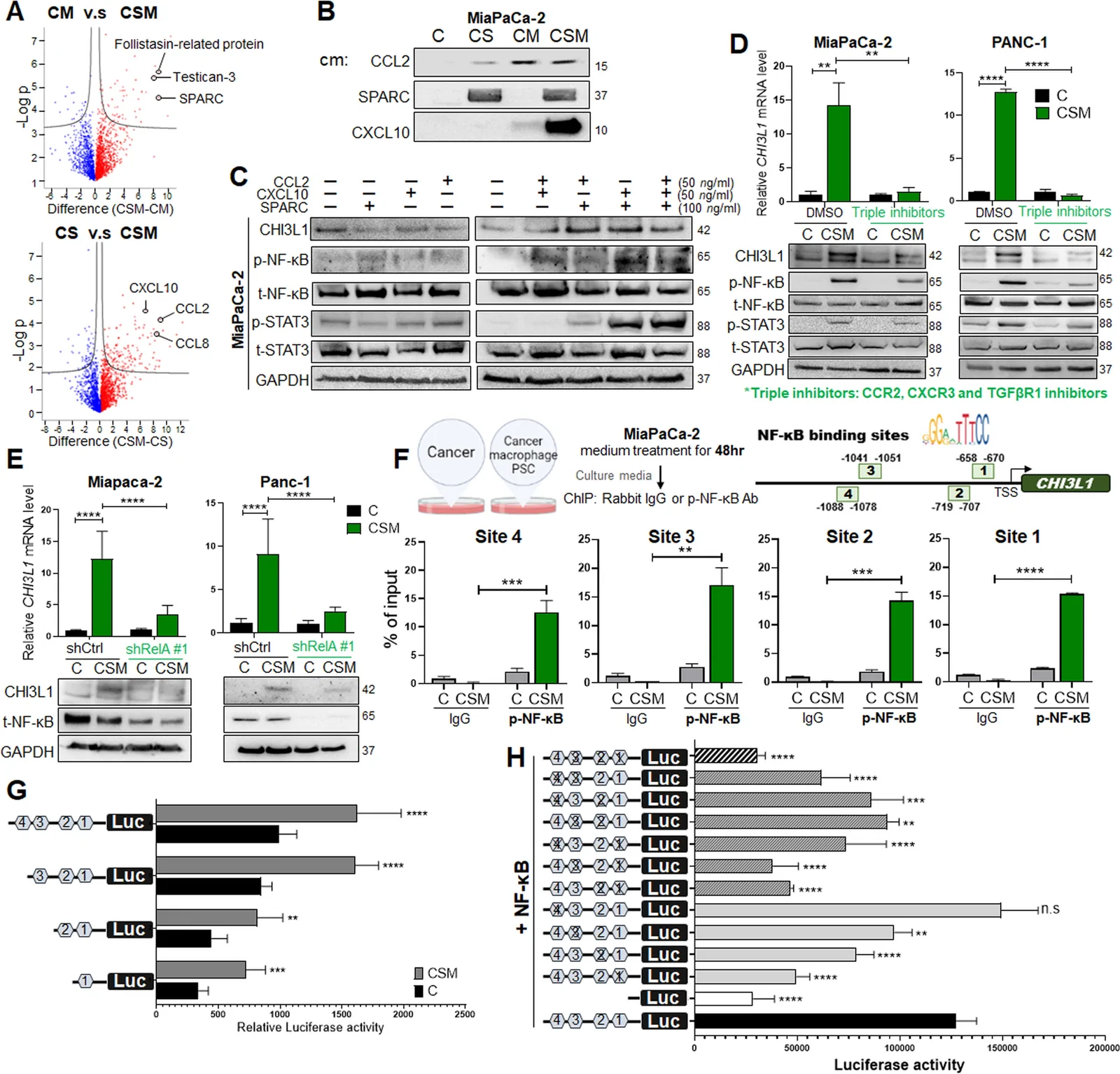
Proteomic and functional dissection of mixed-culture media reveals key CHI3L1-inducing factors. **A** Volcano plots of differentially expressed proteins derived from culture media as indicated. Six secreted proteins were selected. **B** Secreted levels (culture media, cm) of CCL2, SPARC, and CXCL10 proteins were validated in mixed-culture media by immunoblotting. **C** Protein levels of CHI3L1, phosphorylated (p)-NF-κB, and p-STAT3 in MiaPaCa-2 cells with 48 h treatment of recombinant CCL2 (50 *n*g/ml), CXCL10 (50 *n*g/ml), and/or SPARC (100 *n*g/ml). **D** CHI3L1 expression levels in MiaPaCa-2 and PANC-1 were assessed after treatment with or without triple inhibitors targeting CCR2, CXCR3, and TGFβR1, and culturing with C or CSM media. **E** CHI3L1 expression levels in *RelA* knockdown (shRelA) MiaPaCa-2 or PANC-1 cells educated with C or CSM media for 48 h. **F** ChIP-qPCR detection of p-NF-κB binding to the *CHI3L1* promoter in MiaPaCa-2 cells treated with C or CSM media (upper left). Promoter structure of the *CHI3L1* gene. The green rectangle represented the NF-κB binding motif (upper right). Quantitative PCR amplification was performed with DNA fragments immunoprecipitated with anti-IgG or anti-p-NF-κB antibody. **G** MiaPaCa-2 was transfected with pGL4.17-hCHI3L1-NF-κB promoter plasmid containing NF-κB binding sites 1 to 4 as indicated and educated with C or CSM media. **H** MiaPaCa-2 was co-transfected with NF-κB expressing plasmid and pGL4.17-hCHI3L1-NF-κB wild-type or mutant plasmids as indicated. Data are presented as mean ± SD. *P* values were determined by one-way ANOVA (*P < 0.05, **P < 0.01, ***P < 0.001, ****P < 0.0001)

To identify candidate mediators, we analyzed differentially secreted proteins using volcano plots (Fig. 2A) and unbiased KEGG pathway enrichment analysis (Fig. S2E), focusing on factors enriched in CSM relative to CS or CM to define PSC- or macrophage-derived signals. This approach identified macrophage-derived chemokines (CCL2, CCL8, CXCL10) and the PSC-derived protein secreted protein acidic and rich in cysteine (SPARC) as key candidates (Fig. 2A). CCL2, CXCL10, and SPARC were validated by immunoblotting (Fig. 2B). Moreover, while individual recombinant proteins failed to induce CHI3L1, combined treatments increased CHI3L1 expression in PDAC cells (Fig. 2C, Fig. S2F), a response also observed in lung, esophageal, and colorectal cancer cell lines (Fig. S2G-I).

To assess the requirement of the CCR2, CXCR3, and TGFβR1 signaling in CSM-induced CHI3L1 expression, PDAC cells were subjected to receptor knockdown or pharmacological inhibition of CCR2, CXCR3, and TGFβR1. Combined inhibition or knockdown of all three receptors markedly suppressed CSM-induced CHI3L1 expression, whereas dual receptor inhibition was insufficient (Fig. 2D, Fig. S2J-N). These findings indicate that CCL2, CXCL10, and SPARC function cooperatively to regulate CHI3L1 induction through cancer-stroma-immune cell interactions. To explore whether SPARC engages TGFβR1 at the cell surface, we performed cell-surface co-IP in MiaPaCa-2 and PANC-1 cells using recombinant SPARC and detected that TGFβR1 co-immunoprecipitated with SPARC relative to IgG control (Fig. S2O). This finding indicates that exogenously added SPARC associates with a TGFβR1-containing complex at the cell surface.

### CCL2, CXCL10, and SPARC regulate CHI3L1 expression via NF-κB activation during cancer-stromal cell interaction

CCL2/CCR2 and CXCL10/CXCR3 are established ligand-receptor signaling axes known to promote PDAC progression, and SPARC has separately been reported to modulate TGFβR1-dependent signaling in other contexts, by activating oncogenic pathways, including NF-κB, STAT3, and TGF-β signaling [28–36]. Based on these roles, we examined how these factors regulate CHI3L1 expression using mixed-culture systems and clinical PDAC datasets. Spatial transcriptomic analysis revealed significant enrichment of transcription factor target genes, particularly NF-κB, STAT3, SP1, and JUN, in regions where cancer cells interact with stromal and immune cells compared with cancer-only regions (Fig. S3A). Consistently, TCGA RNA-seq analysis showed that CHI3L1^high^ tumors were enriched for inflammatory and tissue-remodeling pathways, including TNFα/NF-κB, IL-6/STAT3, and TGFβ1 signaling (Fig. S3B). Promoter analyses (PROMO and JASPAR) identified binding sites for NF-κB, STAT3, SP1, and JUN within the *CHI3L1* promoter (Fig. S3C). Functional validation demonstrated that NF-κB uniquely and robustly induced *CHI3L1* mRNA and protein in a time-dependent manner (Fig. S3D-F). We also observed that combined treatment with recombinant CCL2, CXCL10, and SPARC (Fig. 2C, Fig. S2F-I) or CSM stimulation (Fig. 2D, Fig. S3G) increased phospho-NF-κB (p-NF-κB) levels in all cells examined. Conversely, inhibition or knockdown of their respective receptors (CCR2, CXCR3, and TGFβR1) (Fig. 2D, Fig. S2J-N) or *RelA* knockdown (Fig. 2E, Fig. S3H, I) significantly attenuated CSM-induced CHI3L1 expression. These results identify NF-κB as a key downstream effector of cancer-stroma signaling in CHI3L1 regulation.

To confirm direct regulation of CHI3L1 by NF-κB, we performed chromatin immunoprecipitation (ChIP)-qPCR and revealed increased p-NF-κB binding at four putative sites on the *CHI3L1* promoter following CSM treatment (Fig. 2F). To precisely define the functional NF-κB-responsive elements, we performed luciferase reporter assays using *CHI3L1* promoter constructs of varying lengths. CSM stimulation markedly enhanced promoter activity (Fig. 2G). Site-directed mutagenesis demonstrated that NF-κB binding sites 1–3 were essential for this response, as their individual or combined mutation significantly reduced promoter activity, whereas mutation of site 4 had minimal effect (Fig. 2H). Together, these data establish CHI3L1 as a direct NF-κB transcriptional target driven by cancer-stroma interactions in the PDAC microenvironment.

### STAT3 promotes *CHI3L1* promoter demethylation and synergizes with NF-κB to enhance CHI3L1 expression during cancer-stroma interaction

**Fig. 3:**
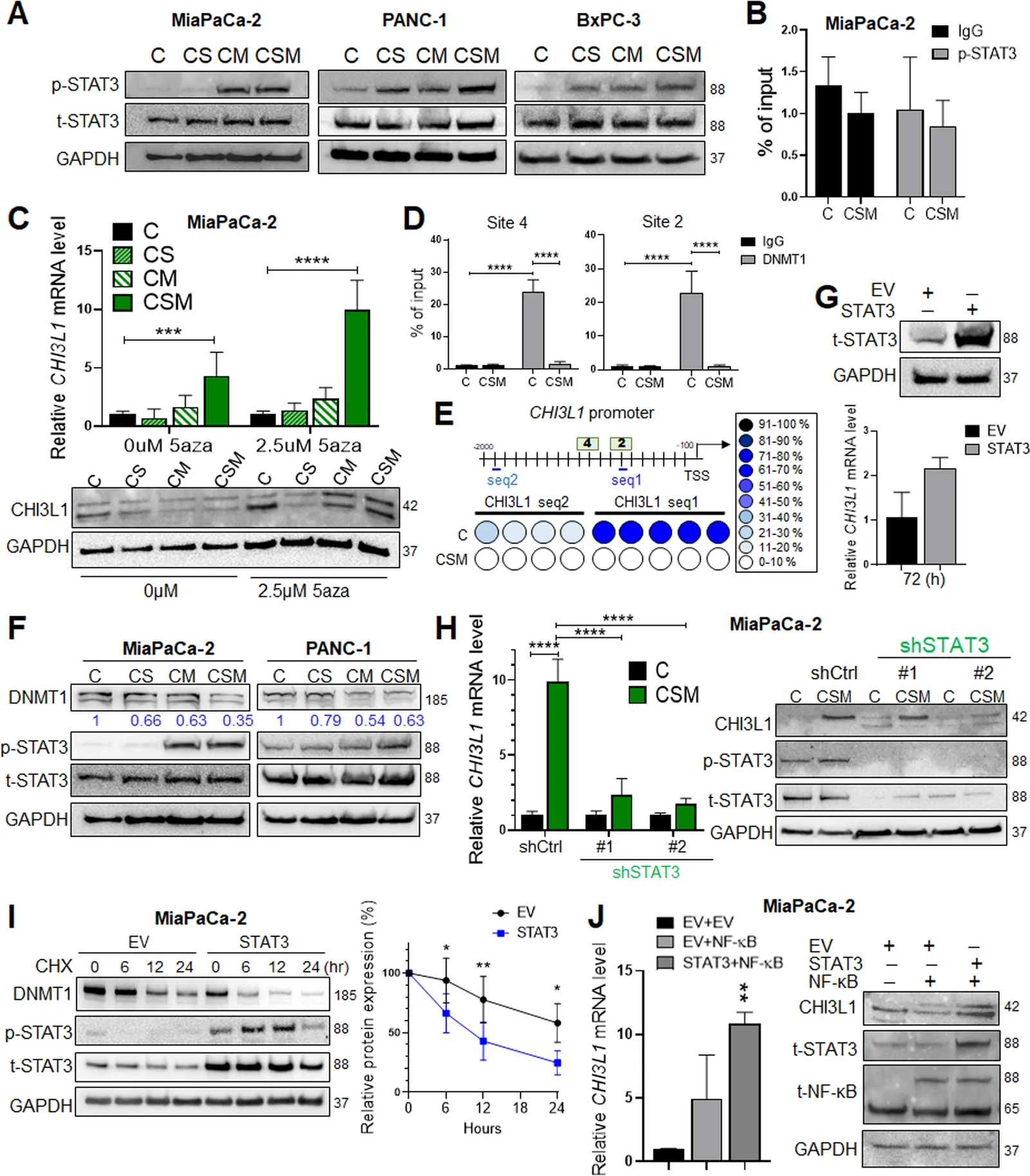
STAT3 activation is required for CHI3L1 induction in cancer cells educated by CSM media. **A** The levels of p-STAT3 in MiaPaCa-2, PANC-1, and BxPC-3 cells educated with C, CS, CM, or CSM media for 48 h. **B** ChIP-qPCR was performed with DNA fragments immunoprecipitated with anti-IgG or anti-p-STAT3 antibody. **C** MiaPaCa-2 cells were pretreated with 2.5 μM 5-azacytidine (5aza) for 72 h, followed by 24 h co-culture media education as indicated. *CHI3L1* levels in MiaPaCa-2 cells were verified by qPCR (upper) and immunoblotting (lower). **D** ChIP-qPCR of DNMT1 occupancy at NF-κB site 2 and site 4 of the *CHI3L1* promoter in C- or CSM-educated PANC-1 cells, immunoprecipitated with anti-IgG or anti-DNMT1 antibody. **E** Bisulfite pyrosequencing quantification of CpG site-specific methylation levels within the *CHI3L1* promoter region in MiaPaCa-2 cells educated with C or CSM conditioned media. The blue color scale indicates percentage methylation (0-100%) across consecutive CpG dinucleotide loci. **F** DNMT1 and p-STAT3 protein levels in MiaPaCa-2 and PANC-1 cells educated with C, CS, CM, or CSM media for 48 h. **G** Validation of STAT3 overexpression by immunoblotting (upper). *CHI3L1* mRNA level in MiaPaCa-2 after 72 h EV or STAT3 transfection (lower). **H** *CHI3L1* mRNA (left) and protein (right) expression levels in shCtrl or shSTAT3 (#1 and #2) MiaPaCa-2 cells educated with C or CSM media for 48 h. **I** Cycloheximide (CHX) chase assay in MiaPaCa-2 cells transfected with EV or STAT3. DNMT1 protein levels were assessed at 0, 6, 12, and 24 h after CHX treatment (left); quantification of relative DNMT1 protein expression over time (right). **J** *CHI3L1* mRNA (left) and protein (right) levels in MiaPaCa-2 cells transfected with STAT3 and/or NF-κB. Data are presented as mean ± SD. *P* values were determined by one-way ANOVA (*P < 0.05, **P < 0.01, ***P < 0.001, ****P < 0.0001)

CS, CM, and CSM conditioned media induced robust STAT3 activation in cancer cells (Fig. 3A); however, STAT3 did not directly bind to the *CHI3L1* promoter (Fig. 3B). Given reports linking STAT3 activation to DNA methyltransferase 1 (DNMT1) degradation and DNA demethylation [37], we examined epigenetic regulation of CHI3L1. Treatment with the DNA methyltransferase inhibitor 5-azacytidine (5-aza) significantly increased CHI3L1 expression after CSM education for 24 h (Fig. 3C), supporting a role for DNA methylation in this process. To identify whether DNMT1 mediates this methylation change, we performed DNMT1 ChIP-qPCR. The ChIP amplicons resolve two promoter regions rather than individual motifs. DNMT1 occupied both sites 2 and sites 4 under monoculture (C) conditions, with occupancy markedly reduced following CSM education (Fig. 3D). Notably, these regions encompass all four NF-κB sites, which were functionally required for CSM-induced promoter activity (Fig. 2H), raising the possibility that DNMT1 loss may facilitate NF-κB access to these elements. Consistently, bisulfite pyrosequencing confirmed a loss of CpG methylation across the *CHI3L1* promoter following CSM education (Fig. 3E), in agreement with the methylation-specific PCR results (Fig. S3J).

Consistent with a STAT3-dependent mechanism, cancer cells educated with CS, CM, or CSM media exhibited elevated p-STAT3 together with a progressive decrease in DNMT1 protein levels (Fig. 3F). Functionally, STAT3 overexpression modestly increased *CHI3L1* mRNA expression (Fig. 3G), whereas STAT3 knockdown attenuated CSM-induced CHI3L1 upregulation (Fig. 3H, S3K). Given that CSM education induced STAT3 activation and concomitant DNMT1 loss (Fig. 3F), we tested whether STAT3 activation destabilizes DNMT1 by performing cycloheximide (CHX) chase assays, which showed accelerated DNMT1 decay in STAT3-overexpressing cells at all timepoints (Fig. 3I). Consistently, rescue of DNMT1 expression suppressed CSM-induced CHI3L1 upregulation (Fig. S3L), validating the requirement of DNMT1 loss in this process. Furthermore, sequential overexpression of STAT3 followed by NF-κB enhanced CHI3L1 expression more than NF-κB alone, suggesting synergistic regulation (Fig. 3J). Collectively, these findings indicate that STAT3 contributes to CHI3L1 induction via promoter demethylation and acts in concert with NF-κB to promote CHI3L1 expression in response to TME-mimicking stimuli (CCL2, CXCL10, and SPARC) in the CSM model (also see Fig. 2C, D, Fig. S2F–N).

### Single-cell transcriptomic analysis identifies CHI3L1-responsive cancer cell subtype driven by macrophage and fibroblast interactions in PDAC

**Fig. 4:**
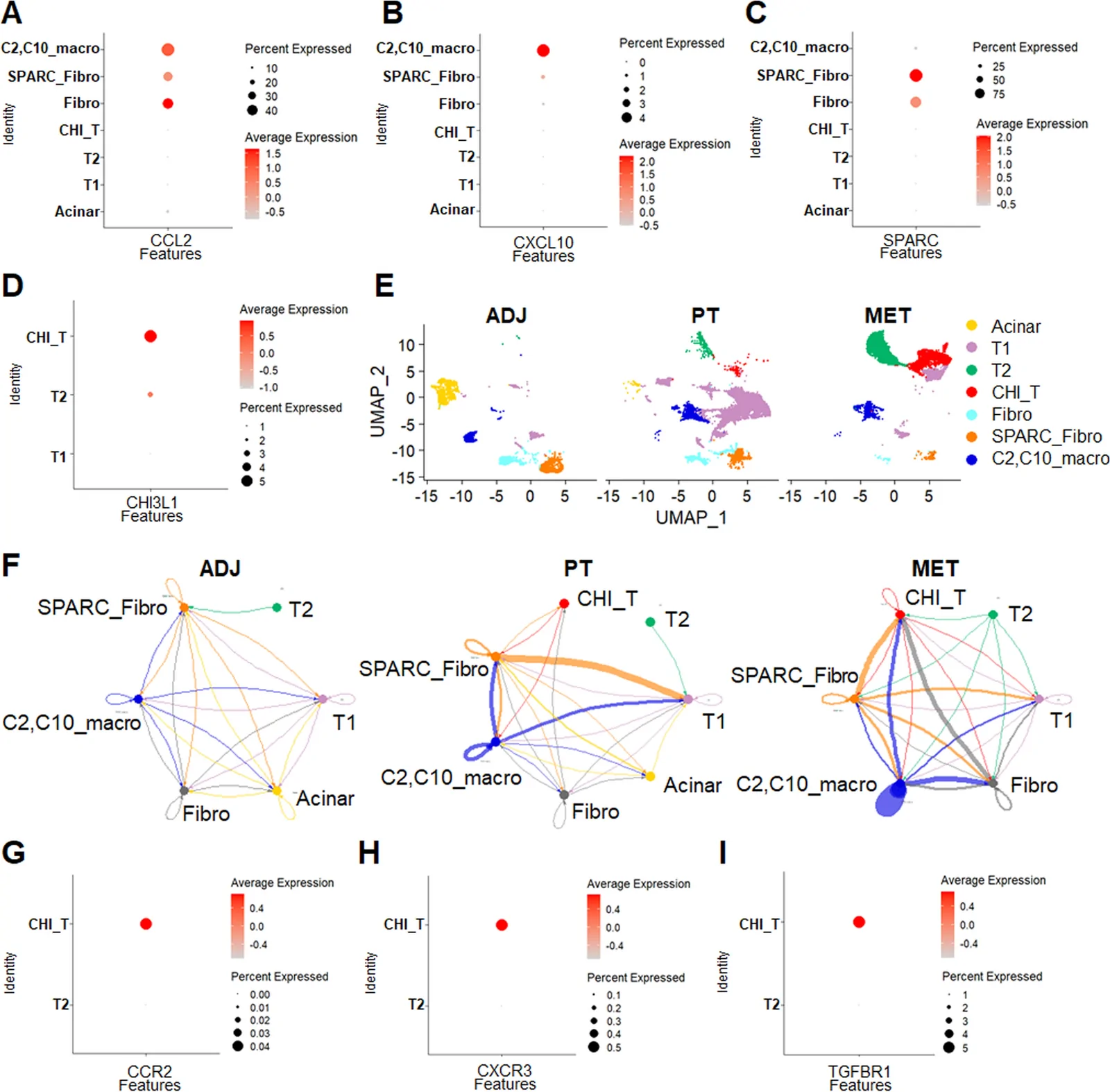
Single-cell profiling identifies a CHI3L1^high^ cancer cell population responsive to macrophage- and fibroblast-derived signals. **A–C** A dot-plot showing the relative expression of *CCL2* (**A**), *CXCL10* (**B**), and *SPARC* (**C**) in all clusters. **D** A dot-plot showing the relative expression of *CHI3L1* in different clusters of tumor cells, T1, T2, and CHI_T. **E** UMAP plots of CHI_T (red) in distinct populations in ADJ, PT, and MET samples. **F** Predicted cell communication network of the seven main cell types in ADJ, PT, and MET samples. **G–I** A dot-plot showing the relative expression of *CCR2* (**G**), *CXCR3* (**H**), and *TGFBR1* (**I**) in CHI_T and T2

To validate our findings in patient samples, we integrated six pancreatic single-cell RNA-seq datasets comprising adjacent normal (ADJ), primary tumor (PT), and metastatic (MET) samples (GSE154778 and GSE205013) [38, 39]. Unsupervised, graph-based clustering (UMAP) identified 14 distinct cellular clusters (Fig. S4A-D), among which macrophages were the predominant source of CCL2 and exclusive producers of CXCL10 (C2,C10_macro cluster; Fig. 4A, B), fibroblasts subdivided into SPARC_Fibro and Fibro populations (Fig. 4C), and cancer cells formed three subclusters, with the CHI3L1^high^ subcluster termed CHI_T (Fig. 4D). Pseudotime analysis, rooted at the T1 population, was consistent with a transcriptional continuum originating from T1 through T2 toward the CHI_T population (Fig. S4E). Given the cross-sectional nature of this dataset, this ordering reflects transcriptional similarity rather than a confirmed temporal or differentiation sequence. Notably, CHI_T (red) was present in PT samples and reached its highest proportion in MET samples (Fig. 4E).

Predicted intercellular communication scores indicated increasing signaling from C2,C10_macro and SPARC_Fibro toward CHI_T from ADJ to PT to MET samples (Fig. 4F), and CCR2, CXCR3, and TGFBR1 were preferentially expressed in CHI_T relative to T2 (Fig. 4G–I), consistent with its responsiveness to macrophage- and fibroblast-derived signals.

### Spatial and functional analyses confirm that CCL2, CXCL10, and SPARC cooperatively regulate CHI3L1 induction in PDAC

**Fig. 5:**
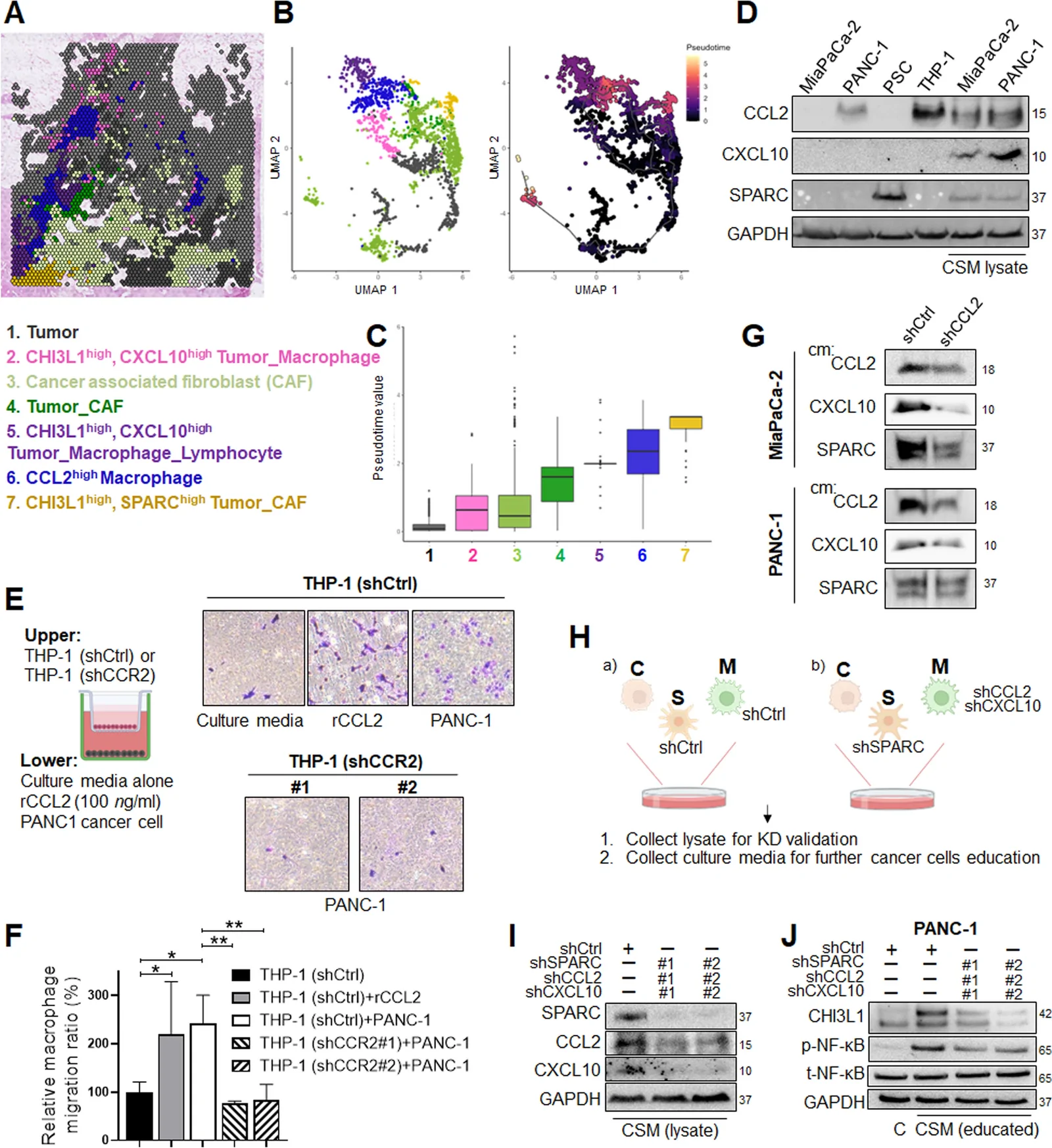
Integrated analysis of CHI3L1 regulatory signals in the PDAC TME. **A** Spatial distribution of CCL2, CXCL10, SPARC, and CHI3L1 expression in PDAC tissues from dataset GSE235315, with each cell population represented by distinct colors. **B** Monocle3 trajectory and pseudotime analysis of the spatial clusters shown in (A), using the tumor-only cluster as the trajectory root; cell-type colors and cluster positions correspond to those in (A). Spots are colored by pseudotime value, with the inferred trajectory graph shown as a solid line. **C** Pseudotime progression across spatial cell populations, ordered into seven groups, corresponds to those in (A) from lowest to highest median pseudotime. **D** Protein levels of CCL2, CXCL10, and SPARC in lysates from MiaPaCa-2, PANC-1, PSCs, and THP-1 cells under monoculture or CSM triple-culture conditions. **E** Transwell assay assessing macrophage recruitment. PMA-activated shCtrl or shCCR2 THP-1 cells were placed in the upper chamber and cultured with medium alone, rCCL2 (100 *n*g/ml), or PANC-1 cells in the lower chamber. Representative images shown. **F** Quantification of transwell migration assays. **G** Secreted CCL2, CXCL10 and SPARC in culture media collected from CSM triple-culture conditions with or without *CCL2* knocked down in THP-1. **H** Schematic of triple-culture models: (a) cancer cell (C) + shCtrl-PSCs (S) + shCtrl-macrophages (M); (b) C + shSPARC-S + shCCL2/shCXCL10-M. **I** Validation of SPARC, CCL2, and CXCL10 knockdown (clones #1 and #2 for each gene) in CSM lysates. **J** CHI3L1 protein levels in PANC-1 cells exposed to C, control CSM, or CSM derived from shCCL2/shCXCL10-M and shSPARC-S. Data are mean ± SD; *P* values determined by one-way ANOVA (*P < 0.05; **P < 0.01)

To investigate whether spatial proximity to CCL2-, CXCL10-, or SPARC-expressing stroma cells contributes to CHI3L1 expression in tumor cells, we analyzed a publicly available spatial transcriptomic dataset (GSE235315) [40], and classified the spots into different clusters [41–46] (Fig. S5A). Clusters with high CHI3L1 expression frequently co-localized with stromal populations expressing CCL2, CXCL10, or SPARC. Specifically, CHI3L1^high^, CXCL10^high^ Tumor_Macrophage (pink); CCL2^high^ Macrophage (blue); CHI3L1^high^, CXCL10^high^ Tumor_Macrophage_Lymphocyte (purple); and CHI3L1^high^, SPARC^high^ Tumor_CAF (yellow), were enriched in each other’s spatial neighborhoods (Fig. 5A). Notably, CHI3L1 expression was significantly higher in the three CHI3L1-high clusters than in Tumor or Tumor_CAF clusters, with no significant difference in CHI3L1 expression among the three CHI3L1-high clusters themselves (Fig. S5B).

Spatial pseudotime analysis indicated an ordering from tumor-only spots through the CHI3L1^high^, CXCL10^high^ Tumor_Macrophage niche and CAF-containing niches at early pseudotime, to Tumor_CAF niches at intermediate pseudotime, and toward the CHI3L1^high^, CXCL10^high^ Tumor_Macrophage_Lymphocyte niche together with CCL2^high^ Macrophage niches at late pseudotime, culminating in the CHI3L1^high^, SPARC^high^ Tumor_CAF niche, which exhibited the highest pseudotime values (Fig. 5B, C). Notably, CXCL10-high niches were represented at both early and late pseudotime, the latter with additional lymphocyte infiltration, suggesting evolving cellular composition rather than a single static state. Given the cross-sectional nature of this dataset, this ordering reflects a continuum of transcriptional similarity across spatial niches rather than a confirmed temporal or differentiation sequence.

We next examined the cellular sources and regulatory mechanisms underlying the elevated levels of CCL2, CXCL10, and SPARC in CSM. Consistent with single-cell analyses, macrophages (THP-1) were the primary source of CCL2, while PSCs were the major producers of SPARC (Fig. 5D). Although single-cell data suggested macrophage-specific CXCL10 expression, CXCL10 was undetectable in monocultures and was induced only under triple-culture CSM conditions (Fig. 5D), indicating that coordinated interactions among cancer cells, stellate cells, and macrophages are required for CXCL10 expression. These results highlight the importance of multicellular crosstalk within the TME in driving the secretion of key CHI3L1-inducing factors.

During tumor progression, elevated levels of inflammatory cytokines, including CCL2, recruit macrophages to the TME [28]. In transwell assays, both recombinant CCL2 (rCCL2, 100 *n*g/mL) and PANC-1 cancer cells (lower chamber) promoted migration of THP-1 cells expressing CCR2, an effect abolished by CCR2 knockdown (Fig. 5E, F, Fig. S5C), confirming that CCL2-CCR2 signaling mediates macrophage recruitment. We next explored whether CCL2 modulates the secretion of CXCL10 and SPARC. Stimulation with rCCL2, but not rCXCL10, robustly upregulated SPARC expression and secretion in PSCs (Fig. S5D), demonstrating that macrophage-derived CCL2 directly and specifically promotes SPARC production in PSCs. Consistent with this, CCL2 depletion in THP-1 macrophages also reduced secreted CXCL10 levels in triple-culture media (Fig. 5G). However, since CCL2 and CXCL10 are co-produced by the same macrophage population, this reduction cannot be attributed to a CCL2-driven induction of CXCL10. These findings reveal a cooperative stroma network: early macrophage recruitment via CCL2 primes PSCs to secrete SPARC, while CXCL10 emerges specifically under multicellular (triple-culture) conditions rather than from any single paracrine axis tested here. Collectively, these factors cooperatively trigger maximal CHI3L1 upregulation in cancer cells.

To determine whether cell–cell interactions and their associated factors are essential for CHI3L1 induction, we generated triple knockdown co-culture systems (Fig. 5H). Specifically, CCL2 and CXCL10 were knocked down in THP-1 macrophages, and SPARC was silenced in PSCs. These modified cells were mixed-cultured with pancreatic cancer cells to produce CSM media from secreted factor-knockdown cells (triple knockdown CSM). Cells with non-targeting controls served as the control CSM (Fig. 5H). Compared with the control, CHI3L1 expression in cancer cells was significantly reduced when cultured with triple knockdown CSM (Fig. 5I, J). In contrast, knocking down CCL2 alone in THP-1 and SPARC in stellate cells did not apparently change the CHI3L1 induction ability of CSM (Fig. S5E-G), indicating that cooperative signaling from all three factors, CCL2, CXCL10, and SPARC, is required for maximal CHI3L1 upregulation.

### CHI3L1/CCL2/CXCL10/SPARC high-expressing profile correlates with PDAC progression in KPPC animal and patient models

**Fig. 6:**
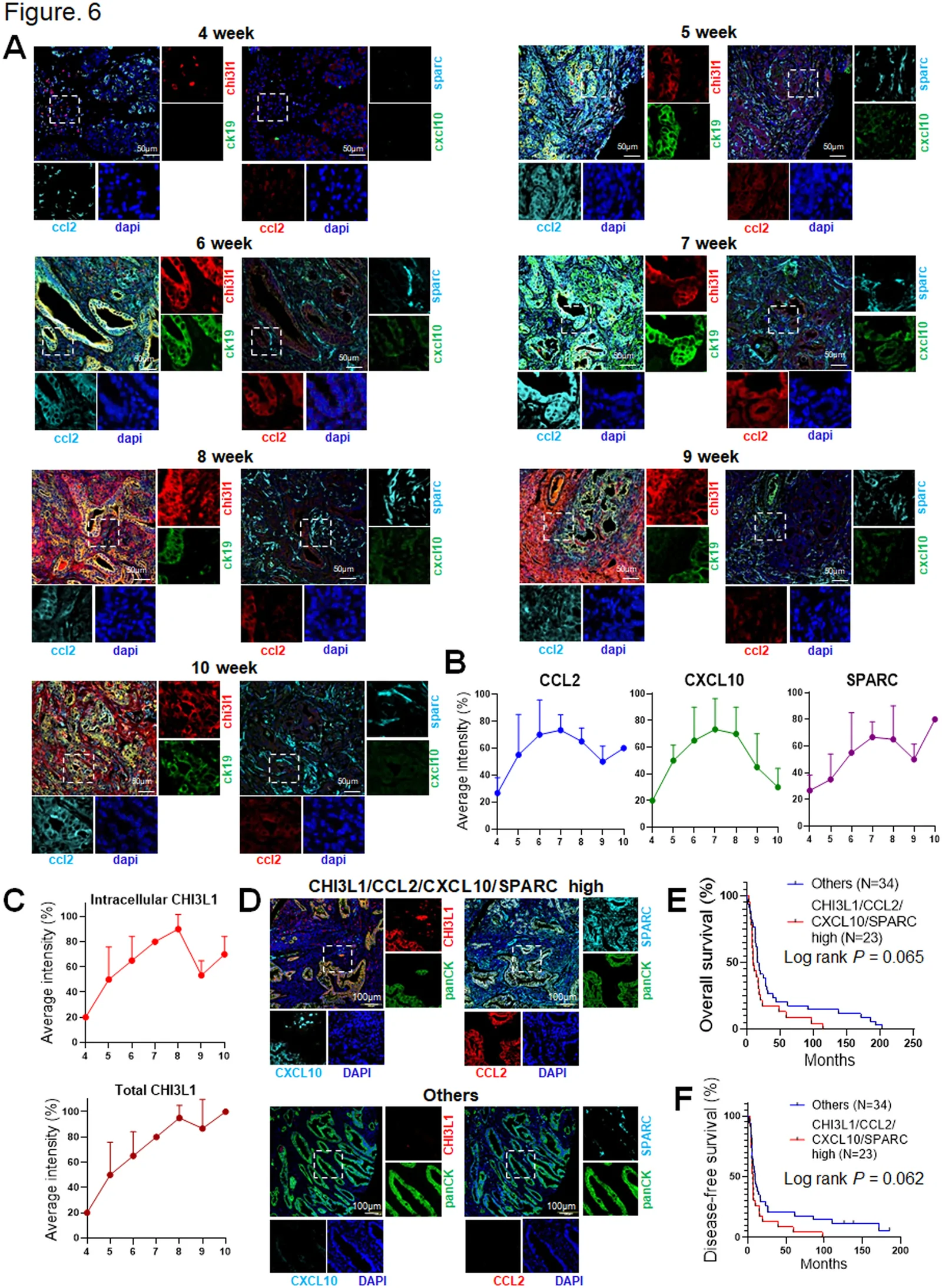
CHI3L1/CCL2/CXCL10/SPARC high-expressing profile correlates with PDAC progression. **A** The expression of chi3l1 (red), ck19 (green), ccl2 (left: light blue; right: red), sparc (light blue), and cxcl10 (green) in two serial sections each of the KPPC transgenic mice at the indicated time (4-10 weeks) was assessed by multiplex IF-IHC (mIF). (Scale bar, 50 μm) **B, C** Statistical results of the average staining intensity of ccl2, cxcl10, sparc (**B**), intracellular chi3l1, and total chi3l1 (**C**). Data are presented as mean ± SD. **D** Representative mIF staining of CHI3L1, panCK, CXCL10, CCL2, and SPARC in PDAC patient tissues from the CHI3L1/CCL2/CXCL10/SPARC four-high (top) and others (bottom) groups (scale bar, 100 μm). Two serial sections were used for staining. **E**, **F** Overall (**E**) and disease-free survival (**F**) in 57 PDAC patients stratified into “CHI3L1/CCL2/CXCL10/SPARC four-high” group (n = 23) and “Others” group (n = 34), assessed by Kaplan–Meier analysis and log-rank test

To examine the expression kinetics of CHI3L1, CCL2, CXCL10, and SPARC, we performed multiplex IF-IHC from the KPPC spontaneous PDAC mouse model [27] collected at weeks 4-10 (n = 3, 4, 4, 3, 4, 4, 2 at weeks 4, 5, 6, 7, 8, 9, and 10, respectively). CCL2 expression increased early (week 5) and remained elevated through week 10, whereas CXCL10 and SPARC peaked at week 7, with SPARC sustaining high levels thereafter (Fig. 6A, B**)**. Intracellular CHI3L1 progressively increased during tumor progression, peaking at weeks 7-8 before declining, while total tissue CHI3L1 levels continued to accumulate (Fig. 6A, C).

Since CCL2, CXCL10, and SPARC are predominantly secreted factors, we further quantified their plasma levels in KPPC mice at weeks 4, 5, 8, 9, and 11 by ELISA (Fig. S6A-C). Plasma SPARC levels significantly increased over time, with significantly higher levels at weeks 8, 9, and 11 compared with week 4 (*P* = 0.005, 0.001, and 0.024, respectively), consistent with the tissue IF kinetics (Fig. 6A, B). Plasma CCL2 and CXCL10 showed a similar increasing trend from week 4 through week 11. Together, these plasma data support a systemic increase in SPARC during PDAC progression, while the corresponding trends for CCL2 and CXCL10 will require larger cohorts to confirm statistically.

Additionally, multiplex staining stratified PDAC patients into CHI3L1/CCL2/CXCL10/SPARC “four-high-expressing group” (n=23) and “others group” (n = 34) (Fig. 6D). Kaplan–Meier analysis showed a trend toward shorter OS (log-rank *P* = 0.065) and DFS (log-rank *P* = 0.062) in the four-high group (Fig. 6E, F), consistent with univariate Cox regression (OS: HR = 1.672, *P* = 0.068; DFS: HR = 1.684, *P* = 0.065). After adjusting for age, T stage, and N stage, multivariable Cox analysis demonstrates that the four-high signature was independently associated with both shorter OS (adjusted HR = 1.852, *P* = 0.040) and with a comparable effect size for DFS that reached borderline significance (adjusted HR = 1.778, *P* = 0.050) (Table 1). Given the modest sample size and borderline significance, validation in a larger cohort is warranted.

**Table 1:** Univariate and multivariable Cox regression analysis of overall survival and disease-free survival in 57 PDAC patients

Characteristics		Overall Survival (OS)				Disease-Free Survival (DFS)			
		Univariate		Multivariable^a^		Univariate		Multivariable^a^	
		HR (95% CI)	*P* value	HR (95% CI)	*P* value	HR (95% CI)	*P* value	HR (95% CI)	*P* value
CHI3L1/CCL2/ CXCL10/ SPARC signature	Others	1		1		1		1	
	Four-high	1.672 (0.963–2.905)	0.068	1.852 (1.029–3.333)	**0.040**	1.684 (0.967–2.932)	0.065	1.778 (1.000–3.160)	**0.050**
Age^b^	Per 1-year increase	1.015 (0.992–1.040)	0.209	1.022 (0.993–1.052)	0.142	1.007 (0.982–1.032)	0.591	1.008 (0.980–1.038)	0.568
T stage	T1–2	1		1		1		1	
	T3	1.470 (0.578–3.739)	0.419	1.229 (0.438–3.452)	0.695	1.409 (0.558–3.560)	0.468	1.260 (0.448–3.544)	0.661
N stage	N0	1		1		1		1	
	N1	1.089 (0.627–1.889)	0.763	0.872 (0.475–1.601)	0.658	1.136 (0.651–1.983)	0.653	0.942 (0.512–1.730)	0.846

HR: hazard ratio; CI: confidence interval

^a^Multivariable model: Cox proportional hazards regression with the four-high signature, age, T stage, and N stage entered simultaneously

^b^Age was entered as a continuous variable rather than dichotomized; the hazard ratio therefore represents the relative change in risk associated with each additional year of age, and no reference category applies

### Simultaneous blockade of TME-inducing pathways and CHI3L1 neutralization remodels the tumor microenvironment and suppresses PDAC progression

CCL2, CXCL10, and SPARC are frequently upregulated in cancers and promote tumor progression and an immunosuppressive TME [30, 32, 34]. Here, we show that these factors cooperatively enhance CHI3L1 expression. We first evaluated the therapeutic potential of combined CCR2 inhibition and CHI3L1 neutralization in an orthotopic human PDAC model. Although dual blockade attenuated tumor growth and fibrosis (Fig. S7A-D), this model lacked an intact adaptive immune system, limiting the ability to fully evaluate anti-tumor immunity and microenvironmental reprogramming. Moreover, targeting CCR2 alone was insufficient to disrupt compensatory signaling from the CXCL10/CXCR3 and SPARC pathways.

**Fig. 7:**
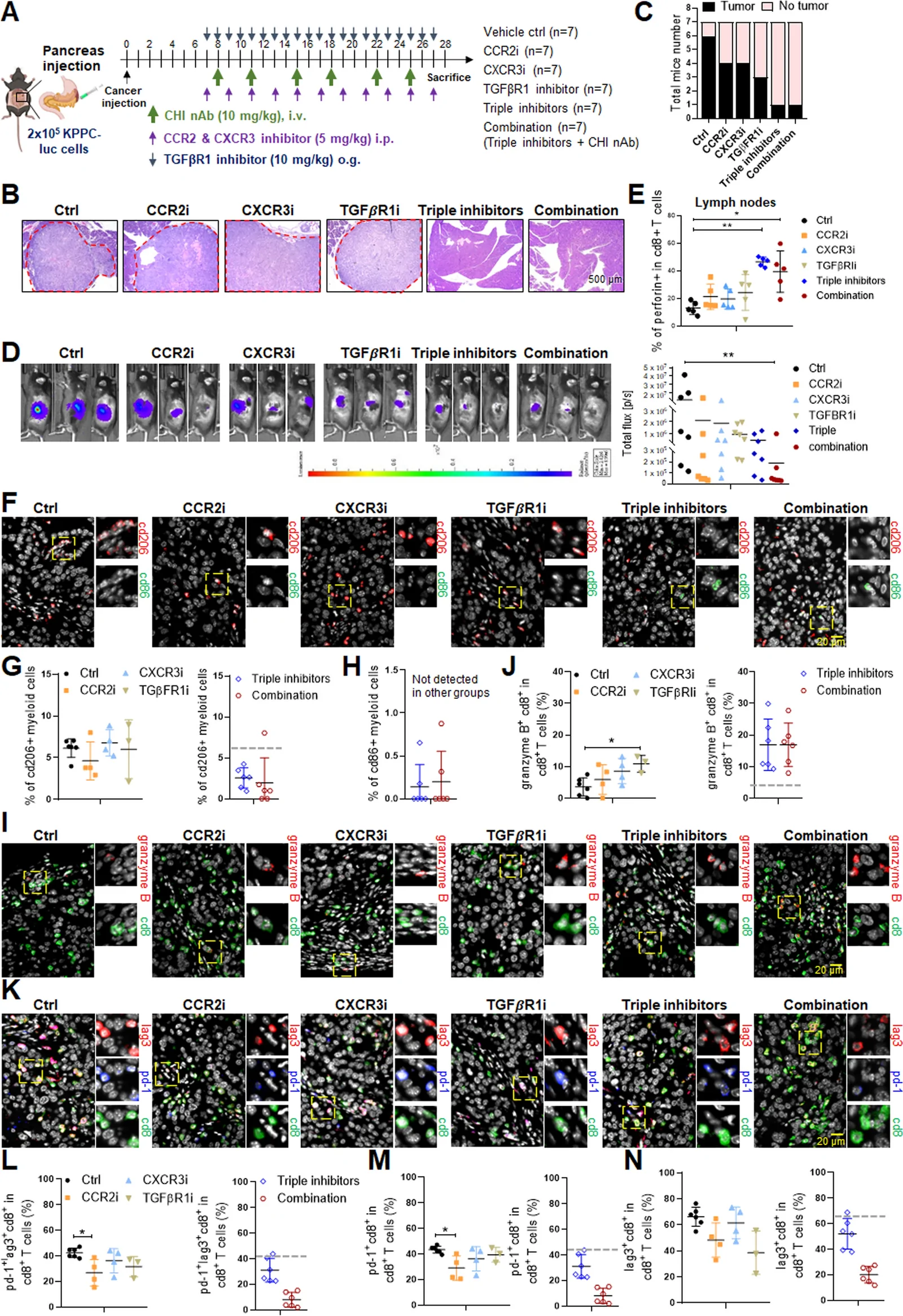
Disrupting the CCR2/CXCR3/TGFβR1 axes together with CHI3L1 neutralization reduces PDAC tumor growth and remodels the tumor immune microenvironment. **A** KPPC-luc cells (2 × 10^5^) were injected into the pancreas of C57BL/6J mice. Experimental design of cancer cell implantation, treatment regimens, collection of blood, and sacrifice. Triple inhibitor group: CCR2 inhibitor (CCR2i, 5 mg/kg), CXCR3 inhibitor (CXCR3i, 5 mg/kg), TGFβR1 inhibitor (TGFβR1i, 10 mg/kg). Combination group: Triple inhibitor plus CHI3L1 nAb (10 mg/kg). (n = 7 for each group) **B** Representative H&E-stained tumor sections across treatment groups; dashed red lines outline the tumor area (Scale bar, 500 μm). **C** Number of mice with a detectable tumor at sacrifice, out of n = 7 per group. **D** Representative IVIS bioluminescence images (left) and quantification (right) of total flux across treatment groups (n = 7 per group). **E** Flow cytometric quantification of the proportions of perforin^+^ cells in cd8 T cells in draining lymph nodes from mice receiving the indicated treatments. **F–H** Representative multiplex IF-IHC (mIF) images (**F**) and quantification of cd206⁺ (red, **G**) and cd86⁺ (green, **H**) myeloid cells in tumors across the indicated groups. cd86⁺ myeloid cells were not detected in the Ctrl, CCR2i, CXCR3i, or TGFβR1i groups (H). Filled symbols represent individual animals with a tumor evaluable at sacrifice (Ctrl, n = 6; CCR2i, n = 4; CXCR3i, n = 4; TGFβR1i, n = 3; see panel **C**), with group means ± SD and statistics in (**G**, **H**, **J**). As only one tumor per group remained evaluable in the triple-inhibitor and combination groups, open symbols instead represent 6 ROIs from that single tumor (technical replicates), plotted against the Ctrl mean (dashed line) for descriptive reference without formal statistical comparison. **I** Representative mIF images of granzyme b⁺cd8⁺ T cells (granzyme b, red; cd8, green) in tumor sections. **J** Quantification of granzyme B⁺cd8⁺ T cells in tumors across treatment groups. **K** Representative mIF images of pd-1⁺lag3^+^cd8⁺ T cells (l pd1, blue; lag3, red; cd8, green) in tumor sections. **L–N** Quantification of pd-1⁺lag3⁺cd8⁺ (**L**), pd-1⁺cd8⁺ (**M**), and lag3⁺cd8⁺ (**N**) T cells in tumor tissues, presented as in (**G**, **H**, **J**). Nuclei were stained with dapi (grey). (Scale bar, 20 μm). Data are presented as mean ± SD. *P* values were determined by one-way ANOVA (*P < 0.05, **P < 0.01, ***P < 0.001, ****P < 0.0001)

To rigorously investigate therapeutic efficacy and immune remodeling in an immunocompetent setting, orthotopic KPPC-luc tumor-bearing C57BL/6J mice were treated with vehicle, single-agent CCR2i, CXCR3i, or TGFβR1i monotherapy, a triple-inhibitor regimen (targeting CCR2, CXCR3, and TGFβR1), or the combination regimen (triple inhibitors combined with a CHI3L1-neutralizing antibody (CHI nAb)) (Fig. 7A). Tumor incidence at sacrifice decreased from 6 of 7 mice in the ctrl group to 4 of 7 (CCR2i and CXCR3i) and 3 of 7 (TGFβR1i), and to 1 of 7 in both the triple-inhibitor and combination groups (Fig. 7B, C), although IVIS flux quantification reached statistical significance only for the combination group versus ctrl (*P* < 0.01; Fig. 7D), with no significant differences among the other pairwise comparisons. αSMA-positive fibrosis was not significantly reduced by any monotherapy relative to ctrl; the triple-inhibitor and combination groups, each represented by a single evaluable tumor, were not included in this statistical comparison (Fig. S7E, F). No treatment-related toxicity was observed across all six groups by serum biochemistry (AST, ALT, albumin, BUN, creatinine) or H&E staining of major organs (Fig. S7G, H).

Flow cytometry of draining lymph nodes showed that both regimens significantly increased perforin⁺cd8⁺ T cells versus ctrl (Fig. 7E, Fig. S7I), while cd86 expression showed a similar but non-significant trend (Fig. S7J, K). Since tumor-infiltrating immune populations could only be assessed in tumors still detectable at the time of sacrifice, this left a single evaluable tumor per group for triple-inhibitor and combination treatments. We therefore present these tumor-level findings (cd206⁺/cd86⁺ myeloid cells, pd-1⁺cd8⁺, lag3⁺cd8⁺, and granzyme B⁺cd8⁺ T cells; Fig. 7F-N) descriptively, plotting individual values against the ctrl mean (dashed line) rather than performing formal statistics. In this comparison, cd206⁺ and granzyme B⁺cd8⁺ populations showed more variable patterns (Fig. 7F–J), while pd-1⁺CD8⁺ and lag3⁺cd8⁺ frequencies in the combination-treated tumor were lower than the ctrl mean (Fig. 7K-N). Collectively, multi-receptor inhibition restricted tumor expansion to the point that there were too few evaluable tumors for formal immune profiling. Lymph-node flow cytometry confirms that both regimens enhance systemic cytotoxic T-cell activity, while the descriptive tumor-level data suggest CHI3L1 neutralization may offer additional immunomodulatory benefit beyond upstream blockade alone, a possibility warranting confirmation in a larger, adequately powered cohort.

## Discussion

**Fig. 8:**
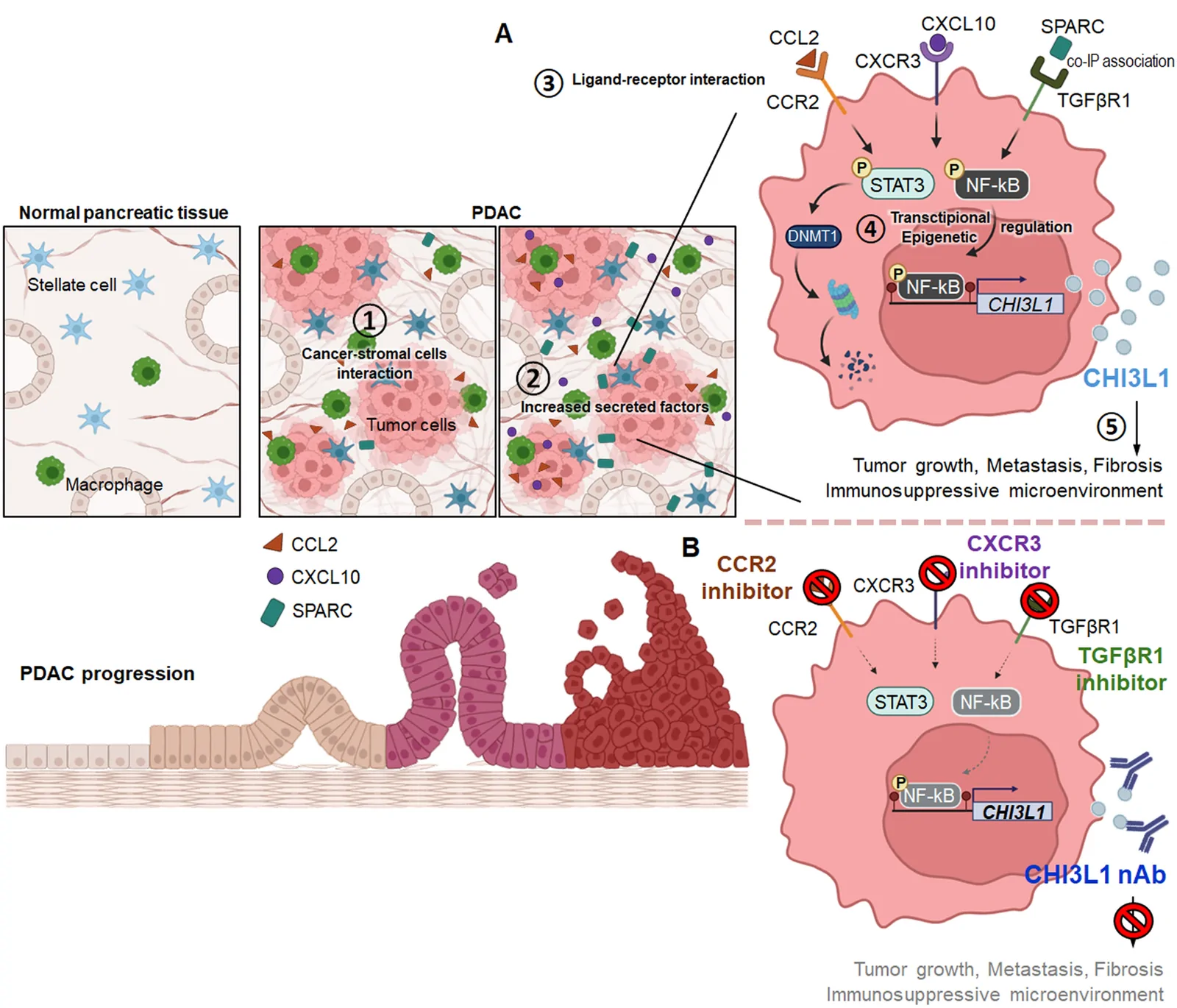
Proposed model depicting cancer-stromal communication and the multi-pathway regulation of CHI3L1 within the tumor microenvironment. **A** ① During tumor progression, inflammatory signals recruit macrophages that produce CCL2, leading to cancer-stromal cell interaction. ② Increased multicellular interactions are associated with enhanced CXCL10 and SPARC secretion. ③ CCL2/CCR2 and CXCL10/CXCR3 signaling, together with TGFβR1-dependent responses to SPARC. ④ activate NF-κB to drive CHI3L1 transcription and are concurrently associated with STAT3-mediated DNMT1 downregulation, resulting in *CHI3L1* promoter hypomethylation and further induction. ⑤ Secreted CHI3L1 promotes tumor growth, metastasis, fibrosis, and immunosuppression. **B** Combining CHI3L1 nAb with CCR2/CXCR3/TGFβR1 inhibition further suppresses CHI3L1-driven tumor-promoting effects

Initially identified in the MG63 osteosarcoma cells [47], CHI3L1 is elevated intracellularly and systemically in multiple cancers, including PDAC [4–13]. In this study, spatial transcriptomics and multicellular co-culture systems revealed that CHI3L1 upregulation in PDAC is not cell-autonomous, but rather driven by coordinated interactions among cancer cells, PSCs, and tumor-associated macrophages. Secretomic and functional studies identified CCL2, CXCL10, and SPARC as cooperative stromal mediators that induce CHI3L1 transcription and promoter hypomethylation via NF-κB binding and STAT3-dependent DNMT1 downregulation, respectively (Fig. 8A). Furthermore, concurrent blockade of these upstream signaling axes (CCR2/CXCR3/TGFβR1) combined with CHI3L1 neutralization reduced tumor burden and was associated with shifts in the tumor immune environment in an immunocompetent PDAC model (Fig. 8B).

At the molecular level, our findings support a cooperative interplay between epigenetic reprogramming and transcriptional activation in driving CHI3L1 expression. Given the activation of NF-κB and STAT3 in response to cancer-stroma interactions and their role in CHI3L1 induction, understanding the underlying regulatory mechanisms is critical. NF-κB activity is regulated through multiple layers, including reversible acetylation of histone and non-histone proteins [48–50]. In parallel, STAT3 activation has been reported to reduce DNMT1 levels and global DNA methylation via JAK/STAT3 signaling [51, 52]. ChIP-qPCR further demonstrated that DNMT1 occupies two sites on the *CHI3L1* promoter encompassing the NF-κB binding elements, with markedly reduced occupancy following CSM education (Fig. 3D-E). Rescue experiments, in which ectopic re-expression of DNMT1 attenuated CSM-induced CHI3L1 expression, support a functional role for DNMT1 loss in this process. Together, these findings establish a dual-layer mechanism, NF-κB-driven transcriptional activation and STAT3-driven epigenetic activation, that promotes robust CHI3L1 induction in cancer cells exposed to complex TME stimuli, providing a mechanistic framework for how chronic stromal inflammation reshapes the cancer epigenome to sustain pro-tumorigenic programs.

A key question raised during the evaluation of TME-induced factors is whether CHI3L1 serves merely as a downstream surrogate marker of stromal activation or contributes actively to disease progression. Multiple studies in solid tumors have established that ectopic overexpression of CHI3L1 in cancer cells directly accelerates in vivo tumor growth, enhances metastatic colonization, and promotes vascularization and M2 macrophage infiltration, confirming its oncogenic driver capacity [13, 14, 16]. Building upon these established gain-of-function paradigms, our in vivo findings in immunocompetent C57BL/6J mice are consistent with a contributory role for CHI3L1 in shaping the tumor immune microenvironment, complementing but not directly establishing causal necessity in this specific model.

Multi-receptor inhibition (targeting CCR2, CXCR3, and TGFβR1) reduced tumor incidence and increased perforin⁺cd8⁺ T cells in draining lymph nodes (Fig. 7C, E), but did not significantly reduce αSMA⁺ stroma (Fig. S7E, F) or fully restore anti-tumor immunity within tumor tissue (Fig. 7F-N). One possible explanation is that CHI3L1, as a stable secreted glycoprotein, may accumulate within the extracellular matrix reservoir of established tumors, and interrupting upstream synthesis alone may not immediately deplete the pre-existing extracellular protein pool. Consistent with this possibility, the addition of CHI3L1 nAb was associated with further shifts in myeloid polarization (increased cd86⁺, decreased cd206⁺ populations), and lower pd-1^+^lag3^+^cd8^+^ frequencies relative to the ctrl mean in the tumors analyzed (Fig. 7F-N). Given the single evaluable tumor per group in the triple-inhibitor and combination arms, establishing an independent, necessary role for CHI3L1 will require adequately powered comparisons in a larger cohort.

Several additional questions remain to be addressed in future studies. First, while our secretomic filtering focused on major protein candidates (CCL2, CXCL10, SPARC), other enriched factors, such as CCL8 were excluded based on their redundant receptor usage (CCR2) [53] and lower relative potency in preliminary screenings. Second, small molecules (< 3 kDa) and metabolic byproducts may also contribute to CHI3L1 regulation. Previous studies have shown that CHI3L1 expression can be modulated by microRNAs, including miR-125a-3p in lung cancer and miR-449a via NOTCH signaling in HCV infection [54, 55]. Additionally, metabolites have emerged as regulators of CHI3L1. For example, lactate has been reported to increase CHI3L1 expression in stromal cells, and histone lactylation (H3K18la) enhances CHI3L1 under metabolic stress [56, 57]. Third, our cell-surface co-IP data (Fig. S2O) indicate that SPARC associates with a TGFβR1-containing complex, but it cannot rule out the possibility that this association is mediated by endogenous cell-surface TGFβ or other intermediary proteins, rather than reflecting direct SPARC-TGFβR1 receptor engagement. Resolving this would require cell-free binding assays using purified proteins. These findings collectively warrant further investigation into the non-proteinaceous and receptor-level components of the TME that may cooperate to modulate CHI3L1 expression.

We further observed that mixed-culture conditioned media not only increased total CHI3L1 levels in cancer cells but also induced higher-molecular-weight CHI3L1 species on immunoblotting. Similar banding patterns have been reported in articular chondrocytes, although their basis remains unclear [58]. Such molecular-weight shifts are typically associated with post-translational modifications (PTMs); however, the PTM landscape of CHI3L1 is poorly characterized, with only a single phosphorylation site (Ser230) reported to date [59]. As a secreted glycoprotein, CHI3L1 undergoes *N*-glycosylation, which is critical for its interaction with IL-13Rα2 [60]. Consistently, mutation of the *N*-glycosylation site abolished the higher-molecular-weight forms (data not shown), implicating glycosylation as a major contributor. Future studies are needed to define the specific modifications involved and to determine whether these CHI3L1 variants differ in localization, stability, or tumor-promoting functions.

## Conclusions

Our study uncovers a multi-layered regulatory network in which stromal CCL2, CXCL10, and SPARC contribute to CHI3L1 expression in PDAC through transcriptional and epigenetic mechanisms. Combined targeting of upstream stromal receptor axes suppresses tumor growth, and the addition of CHI3L1 neutralization was associated with a further reduction in markers of T-cell exhaustion in the tumors analyzed, suggesting a potentially promising combinatorial strategy for PDAC that warrants validation in larger, adequately powered studies. More broadly, the crosstalk we describe between stromal-derived factors and cancer cell receptor-mediated epigenetic and transcriptional upregulation may offer a useful framework for other proteins that exhibit stepwise upregulation during tumorigenesis.

## Supplementary Information

Supplementary material 1.

## Data Availability

All data generated in this study are available from the corresponding author upon reasonable request.

## Abbreviations

PDAC: Pancreatic ductal adenocarcinoma
TME: Tumor microenvironment
CAF: Cancer-associated fibroblasts
PSC: Pancreatic stellate cells
CHI3L1: Chitinase-3-like-protein 1
IL-13Rα2: Interleukin-13 receptor alpha-2
Treg: Regulatory T cell
TF: Transcription factor
GBM: Glioblastoma
hPBMC: Primary human peripheral blood mononuclear cell
PMA: Phorbol 12-myristate 13-acetate
C: Cancer cells culture media
CS: Cancer cells and PSCs double-culture media
CM: Cancer cells and macrophages double-culture media
CSM: Cancer cells PSCs and macrophages triple-culture media
ChIP: Chromatin immunoprecipitation
MSP: Methylation-specific PCR
ROI: Regions of interest
panCK: Pan-cytokeratin
CK19: Cytokeratin 19
PSR: Picro-sirius red
KPPC: *LSL-Kras*^*G12D*^^/+^; *Trp53*^*fl*^^/^^*fl*^; *Pdx1-Cre*
5-aza: DNA methyltransferase inhibitor 5-azacytidine
DNMT1: DNA methyltransferase 1
ADJ: Adjacent normal
PT: Primary tumor
MET: Metastatic samples
