# Supplementary material for: Stromal CCL2/CXCL10/SPARC axes drive pro-tumor CHI3L1 expression for microenvironmental modulation in pancreatic cancer progression and therapeutic innovation
Source: J Biomed Sci. 2026 Oct 26;33:93. doi: 10.1186/s12929-026-01295-2 (PMC13711852; doi:10.1186/s12929-026-01295-2)
Supplement: Supplementary file 1 [file 12929_2026_1295_MOESM1_ESM.pdf]

Supplementary information for

Stromal CCL2/CXCL10/SPARC axes drive pro-tumor CHI3L1 expression for microenvironmental modulation in pancreatic cancer progression and therapeutic innovation

Pei-Chia Su, Yan-Shen Shan, Ya-Chin Hou, Po-Hsien Huang, Pao-Chi Liao, Yi-Ching Wang

This PDF file includes:

Supplementary Materials and Methods

Figure S1 is related to Figure 1

Figure S2 is related to Figure 2

Figure S3 is related to Figures 2 and 3

Figure S4 is related to Figure 4

Figure S5 is related to Figure 5

Figure S6 is related to Figure 6

Figure S7 is related to Figure 7

Table S1 is related to Materials and Methods.

Table S2 is related to Materials and Methods.

Table S3 is related to Materials and Methods.

Supplementary Materials and Methods

Immunoblotting

MiaPaCa-2, PANC-1, BxPC-3, H1299, KYSE510, HCT116, PSC, or THP-1 cells were harvested and lysed in 1 x RIPA buffer containing 1 x protease inhibitors cocktail (Sigma-Aldrich, Saint Louis, USA) and 1 mM DTT (Sigma-Aldrich). Cell lysates were centrifuged at 13,200 rpm for 10 min at 4°C. Protein extracts were solubilized in loading buffer (Millipore). A total of 50 μg of protein lysates were loaded onto 8% sodium dodecyl sulfate/polyacrylamide gel electrophoresis (SDS-PAGE) and electro-transferred onto a polyvinyl difluoride (PVDF) membrane. The membrane was blocked for at least 10 min with 5% non-fat milk and hybridized with the indicated primary antibodies overnight at 4°C. The next day, the membrane was washed and incubated with secondary antibodies. Immunoreactive proteins were visualized using Western blot chemiluminescence reagent (Millipore), and the signals were detected by Luminescence Readers (FUJI LAS-1000, Fujioka, Japan). The antibodies used and the experimental conditions are listed in **Table S1**.

RNA extraction and quantitative reverse transcription PCR (RT-qPCR)

Total RNA was extracted using REzol™ C & T reagent (PROtech, Taipei, Taiwan). Total RNA (1 μg) was reverse transcribed using PrimeScript RT reagent Kit (TaKaRa Bio, Noji higashi, Japan) in a PCR machine (PTC-200 Peltier Thermal Cycler, MJ Research, Quebec, Canada) to generate cDNA for real-time PCR. Real-time PCR was performed to analyze the *CHI3L1* mRNA expression by using the StepOnePlus Real-Time PCR system (Applied Biosystems, Carlsbad, CA, USA) with *β-actin* as an internal control. The cDNA of samples was amplified using the Fast SYBR Green Master Mix (ABclonal Technology, MA, USA) with primers specific to the target genes and thermal cycling conditions as described in **Table S2**. The concentration ratios of target genes were calculated using $2^{-\triangle Ct\ (\triangle Ct\ =\ Ct\text{-}CHI3L1\text{-}Ct\text{-}\beta\text{-}actin}$. Data were presented as fold differences relative to target gene expression versus internal control based on calculation of $2^{-\triangle\triangle Ct\ (\triangle\triangle Ct\ =\ \triangle Ct\text{-}CHI3L1\text{-}\triangle Ct\text{-}\beta\text{-}actin)}$. Each data set contains three biological replicates.

Enzyme-linked immunosorbent (ELISA) assay

Peripheral blood was collected from KPPC mice at weeks 4, 5, 8, 9, and 11 via cardiac puncture and centrifuged at 400*g* for 7 min to isolate plasma, which was stored at -80°C until use. CCL2 and CXCL10 were measured using DuoSet ELISA kits (DY479 and DY466, R&D Systems, Minneapolis, MN, USA); SPARC was measured using a complete sandwich ELISA kit (NBP2-68075, Novus Biologicals), according to the manufacturer's instructions. Plasma samples were diluted 1:10 (CCL2), 1:5 (CXCL10), and 1:20 (SPARC) prior to assay. Absorbance was read at 450 nm with 570 nm correction by ELISA reader (VersaMax Microplate Reader). Each test included a standard control. Because CXCL10 required the lowest dilution and less plasma was available from mice at later, more advanced timepoints, the number of animals assayed varied by analyte and timepoint.

Pseudotime trajectory of single-cell analysis

Pancreatic ductal cell pseudotime trajectories were reconstructed using Monocle3 (v1.3.7). A cell_data_set object was constructed using raw SCT counts from Acinar, T1, T2, and CHI_T cells. To maintain spatial consistency, UMAP coordinates pre-calculated in Seurat were imported into Monocle3. After preprocessing (30 dimensions) and clustering (*cluster_cells*), the principal trajectory graph was learned (*learn_graph,* use_partition = FALSE). Pseudotime was calculated by ordering cells along the graph (*order_cells*) with the trajectory root (pseudotime = 0) defined at the T1 population (node 1, bottom-right). Trajectories were visualized using plot_cells. Because pseudotime ordering was derived from cross-sectional single-cell data, it reflects a continuum of transcriptional similarity among cell states rather than a directly confirmed temporal or lineage relationship.

Flow cytometry

To determine the immune profile of tumor-bearing mice, lymph nodes were stained with anti-mouse CD8, CD11b, CD86, and perforin antibodies. All antibodies were diluted to 1:200 with staining buffer. Cells were stained and incubated on ice for 30 min. For the nuclear protein, cells were fixed with 2% paraformaldehyde for 5 min, then incubated in permeabilization buffer for 10 min prior to further

antibody staining. After washing twice, cells were resuspended in staining buffer and analyzed by flow cytometry (CytoFLEX™). The details of antibodies used for flow cytometry analysis are listed in **Table S1**.

Immunochemistry (IHC) assay

Tumor tissues from pancreatic cancer patients were dewaxed and rehydrated through graded ethanol washes. The immunostaining process was performed according to the Max polymer detection system kit (Leica Biosystems, Wetzlar, Germany). Antigen retrieval was done by incubating the slides for 20 min at 100°C. After blocking, the slides were incubated with primary antibodies. The peroxidase activity was visualized using a diaminobenzidine tetrahydroxychloride solution. The sections were counter-stained with hematoxylin. The quantification of DAB-positive staining was performed using a semi-quantitative scoring system based on both chromogen intensity and the percentage of positive cells. The staining intensity was categorized on a scale of 1 to 4. The size of the ROI was 200 x 200 µm (0.04 mm^2) in allografts from mice or PDAC patients. The antibodies used for protein detection are listed in **Table S1**.

Picro-sirius red (PSR) staining

PSR staining was performed to detect the collagen level within the pancreatic tumor. The slides with tumor tissues were heated and soaked in xylene for deparaffinization. The slides were washed with ethanol twice and ddH_2O once. The slides were then soaked in the micro-Sirius solution (Scytek, Logan, UT, USA) and placed on a shaker for one hour. This step was performed in the absence of light. After staining, the slides were washed twice with ddH_2O and once with absolute alcohol. Measurements were performed on more than three non-overlapping ROIs using ImageJ software.

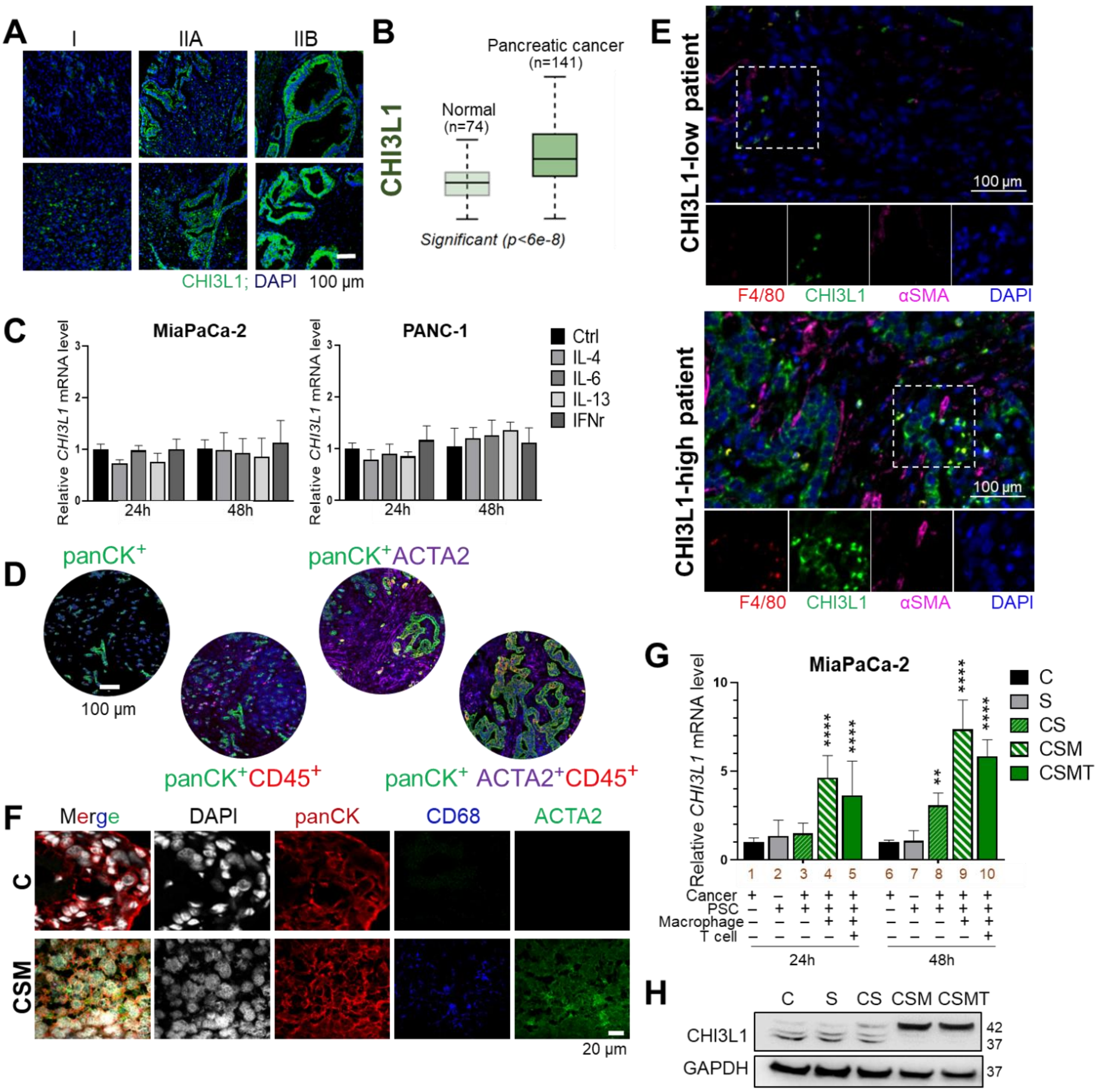

Figure S1. Expression pattern of CHI3L1 in clinical specimens and 3D spheroid cultures. (**A**) Representative images of IF-IHC-stained slides from PDAC patients showing the expression of CHI3L1. (Scale bar, 100 μm) CHI3L1 (green), nucleus (DAPI). (**B**) CHI3L1 protein levels in normal and PDAC tissues from the Human Protein Atlas database. (**C**) *CHI3L1* mRNA level after 24 and 48h of IL-4 (10 *n*g/ml), IL-6 (20 *n*g/ml), IL-13 (10 *n*g/ml), or IFNγ (10 *n*g/ml) treatment in MiaPaCa-2 and PANC-1. (**D**) Representative images of IF-IHC-stained slides from PDAC patients showing the distribution of cancer and various stromal cells for further whole transcriptome analysis (Scale bar, 100 μm). Pan-cytokeratin (panCK; green), CD45 (red), ACTA2 (purple), and Syto13 (blue) are stained for cancer cells, immune cells, fibroblasts, and nuclei, respectively. (**E**) The expression patterns of CHI3L1 (green), F4/80 (red), αSMA (pink), and DAPI (blue) in CHI3L1-low (upper) and CHI3L1-high (lower) PDAC

patient tumor sections were assessed by multiplex IF-IHC. (Scale bar, 100 μm). (**F**) Validation of cancer monoculture (upper) and CSM triple-culture (lower) spheroids by IF-IHC using specific markers. PanCK (red), CD68 (blue), ACTA2/αSMA (green), and DAPI (white) are stained for cancer cells, macrophages, PSCs, and nuclei, respectively. (Scale bar, 20 μm) (**G**) *CHI3L1* mRNA levels in cancer cells educated by culture media collected from C, S, CS, CSM, and/or CSMT mixed culture media with T cells (T). (**H**) CHI3L1 protein levels after 48h treatment of the indicated different mixed culture media. Data are presented as mean ± SD. P values were determined by one-way ANOVA (** $P < 0.01$, **** $P < 0.0001$).

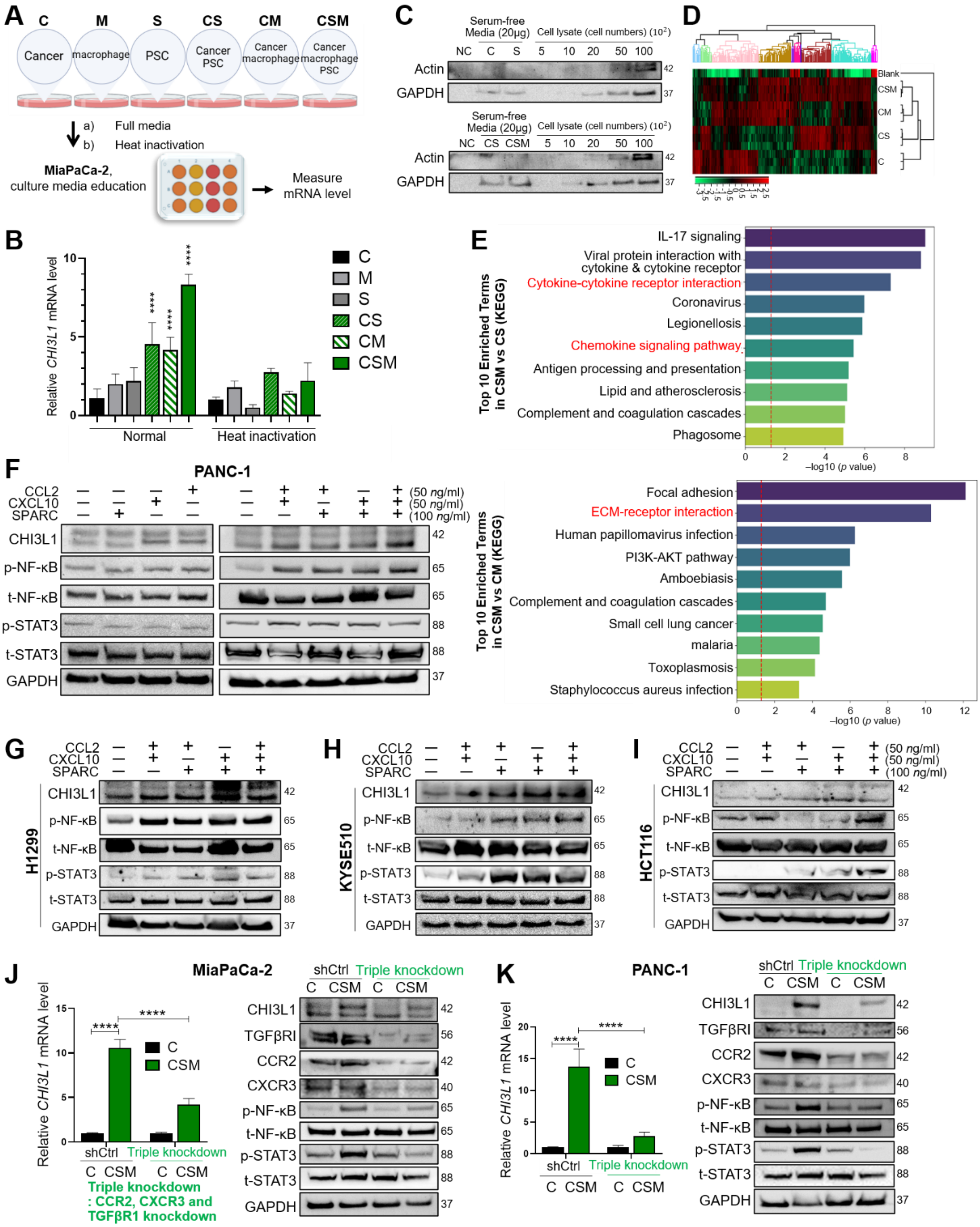
A
C
M
S
CS
CM
CSM
Cancer
macrophage
PSC
Cancer PSC
Cancer macrophage
Cancer macrophage PSC
a) Full media
b) Heat inactivation
MiaPaCa-2,
culture media education
Measure mRNA level
B
Relative CHI3L1 mRNA level
Normal
Heat inactivation
C
M
S
CS
CM
CSM
C
Serum-free Media (20μg)
Cell lysate (cell numbers) (10²)
NC
C
S
5
10
20
50
100
Actin
GAPDH
Serum-free Media (20μg)
Cell lysate (cell numbers) (10²)
NC
CS
CSM
Actin
GAPDH
D
Blank
CSM
CM
CS
C
E
Top 10 Enriched Terms in CSM vs CS (KEGG)
IL-17 signaling
Viral protein interaction with cytokine & cytokine receptor
Cytokine-cytokine receptor interaction
Coronavirus
Legionellosis
Chemokine signaling pathway
Antigen processing and presentation
Lipid and atherosclerosis
Complement and coagulation cascades
Phagosome
–log10 (p value)
Top 10 Enriched Terms in CSM vs CM (KEGG)
Focal adhesion
ECM-receptor interaction
Human papillomavirus infection
PI3K-AKT pathway
Amoebiasis
Complement and coagulation cascades
Small cell lung cancer
malaria
Toxoplasmosis
Staphylococcus aureus infection
–log10 (p value)
F
PANC-1
CCL2 (50 ng/ml)
CXCL10 (50 ng/ml)
SPARC (100 ng/ml)
CHI3L1
p-NF-κB
t-NF-κB
p-STAT3
t-STAT3
GAPDH
G
H1299
H
KYSE510
I
HCT116
CCL2
CXCL10
SPARC
(50 ng/ml)
(50 ng/ml)
(100 ng/ml)
CHI3L1
p-NF-κB
t-NF-κB
p-STAT3
t-STAT3
GAPDH
J
MiaPaCa-2
Relative CHI3L1 mRNA level
C
CSM
shCtrl
Triple knockdown
Triple knockdown
: CCR2, CXCR3 and
TGFβR1 knockdown
CHI3L1
TGFβRI
CCR2
CXCR3
p-NF-κB
t-NF-κB
p-STAT3
t-STAT3
GAPDH
K
PANC-1
Relative CHI3L1 mRNA level
shCtrl
Triple knockdown
C
CSM
CHI3L1
TGFβRI
CCR2
CXCR3
p-NF-κB
t-NF-κB
p-STAT3
t-STAT3
GAPDH

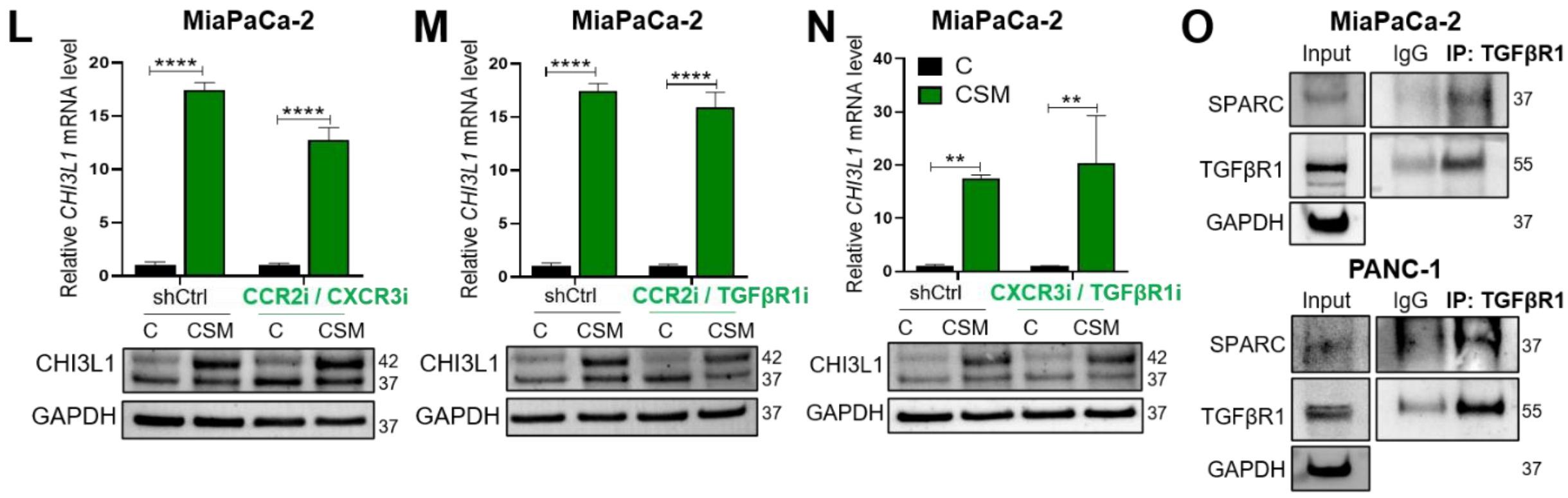

Figure S2. CSM-activated CCR2, CXCR3, and TGFβR1 signaling cooperates to induce CHI3L1 expression. (**A**) Model of experimental design with or without heat-inactivated (100°C for 10 min) culture media. (**B**) *CHI3L1* mRNA levels in MiaPaCa-2 cells educated with full or heat-inactivated culture media. (**C**) Cell lysis rate was determined by comparing the Actin and GAPDH levels in mixed culture media collected under serum-free conditions with those in cell lysates from cultures containing defined cell numbers. (**D**) Hierarchical clustering heatmap of proteins identified in culture media collected from defined culture media (C, CS, CM, and CSM) using mass spectrometry. In the heatmap, green and red indicate decreased and increased compound abundance, respectively. (**E**) Top 10 enriched KEGG pathways for proteins upregulated in CSM vs. CS (left) and CSM vs. CM (right) secretomic dataset. Dashed red lines denote the statistical significance threshold $-\log_{10}(p) > 1.3$, corresponding to $P = 0.05$. (**F-I**) Protein levels of CHI3L1, p-NF-κB, and p-STAT3 in PANC-1 (F), H1299 (G), KYSE510 (H), and HCT116 (I) with 48h mono- or combined treatment of recombinant CCL2 (50 *n*g/ml), CXCL10 (50 *n*g/ml), and/or SPARC (100 *n*g/ml). (**J-K**) Expression levels of CHI3L1, p-NF-κB, and p-STAT3 in MiaPaCa-2 (J) and PANC-1 (K) cells were assessed after treatment with shCtrl or a triple knockdown of CCR2, CXCR3, and TGFβR1, and culturing with C or CSM media. (**L-N**) Expression levels of CHI3L1 in MiaPaCa-2 cells were assessed after treatment with DMSO or inhibitors of CCR2i (5 *n*M) and CXCR3i (10 *n*M) (L); DMSO or CCR2i (5 *n*M) and TGFβR1i (250 *n*M) (M); DMSO or CXCR3i (10 *n*M) and TGFβR1i (250 *n*M) (N). (**O**) Cell-surface co-IP of recombinant SPARC with TGFβR1 in MiaPaCa-2 (upper) and PANC-1 (lower) cells. Data are presented as mean ± SD. *P* values were determined by one-way ANOVA (** $P < 0.01$, **** $P < 0.0001$).

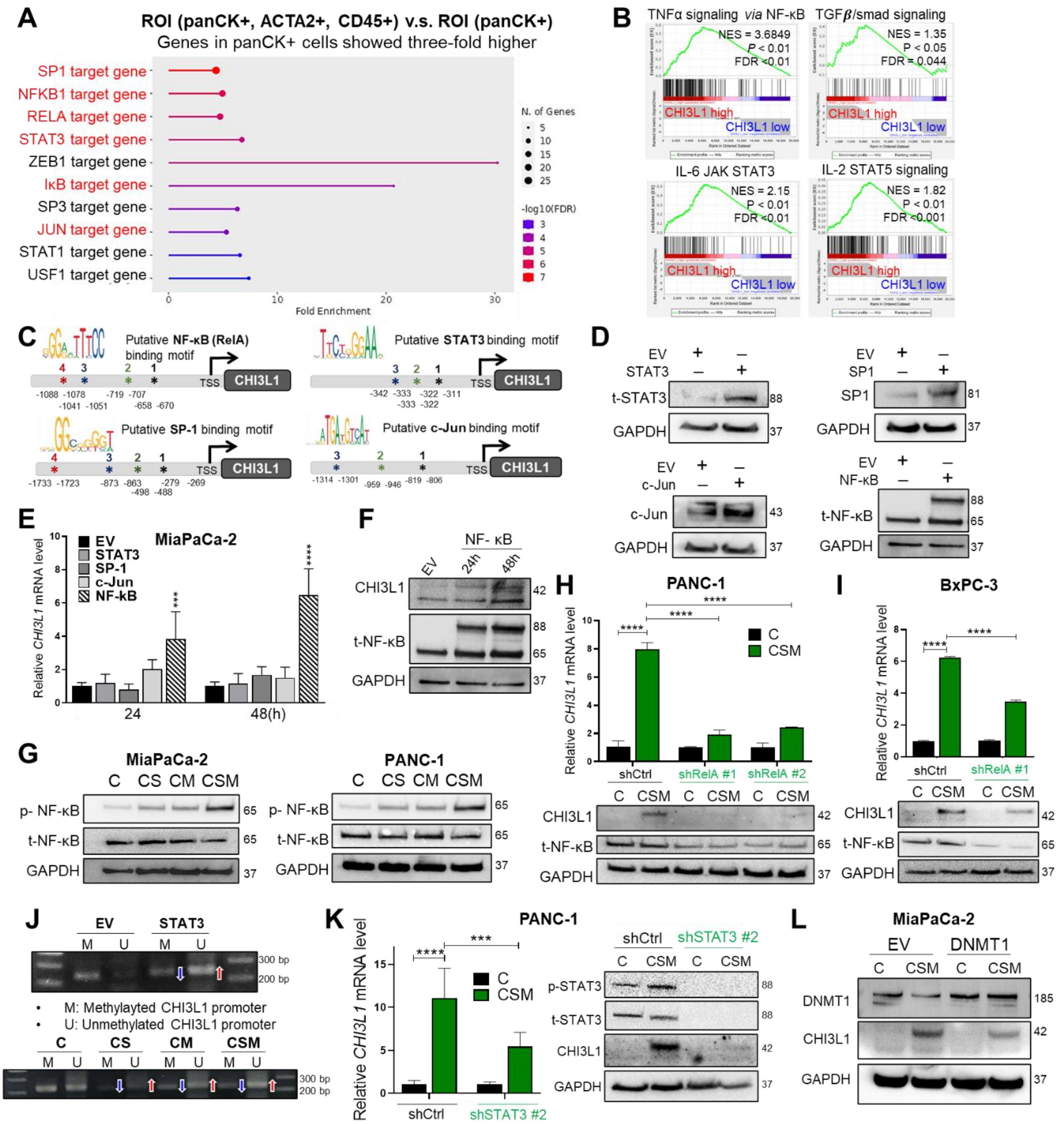

Figure S3. NF-κB regulates CHI3L1 expression within cancer cells. (**A**) Differentially expressed genes of the indicated transcription factors within cancer cells from different regions of interest (ROI): panCK^{+}, ACTA2^{+}, and CD45^{+} versus panCK^{+}. (**B**) GSEA analysis of pathways enriched in pancreatic cancer surgical specimens with high *CHI3L1* expression levels. (**C**) Transcription factors (TFs) and their predicted binding sites on the *CHI3L1* promoter. (**D**) Validation of ectopic overexpressed TFs in MiaPaCa-2 cells by immunoblotting. (**E**) *CHI3L1* mRNA levels in MiaPaCa-2 cells after 24h and 48h transfection with the indicated TFs, including STAT3, SP1, c-Jun, and NF-κB. (**F**) CHI3L1 protein levels increased in a time-dependent manner with the duration of NF-κB transfection. (**G**) The levels of p-NF-κB in

MiaPaCa-2 and PANC-1 cells educated with C, CS, CM, or CSM media for 48h. (**H**) CHI3L1 expression levels in shCtrl or shRelA (#1 and #2) PANC-1 cells educated with C or CSM media for 48h. (**I**) CHI3L1 expression levels in shRelA BxPC-3 cells educated with C- or CSM-media for 48h. (**J**) Bisulfite-modified genomic DNA from empty vector (EV) or STAT3 overexpressed (upper) and C, CS, CM, or CSM-treated (lower) MiaPaCa-2 cells were subjected to MSP analysis to assess the methylation level on the *CHI3L1* promoter. M, methylation-specific reaction; U, unmethylation-specific reaction. (**K**) *CHI3L1* mRNA (left) and protein (right) expression levels in shCtrl or shSTAT3 (#2) PANC-1 cells educated with C or CSM media for 48h. (**L**) DNMT1 and CHI3L1 protein levels in EV- or DNMT1-expressing MiaPaCa-2 cells educated with C or CSM medium. Data are presented as mean ± SD. *P* values were determined by one-way ANOVA (*** $P < 0.001$, **** $P < 0.0001$).

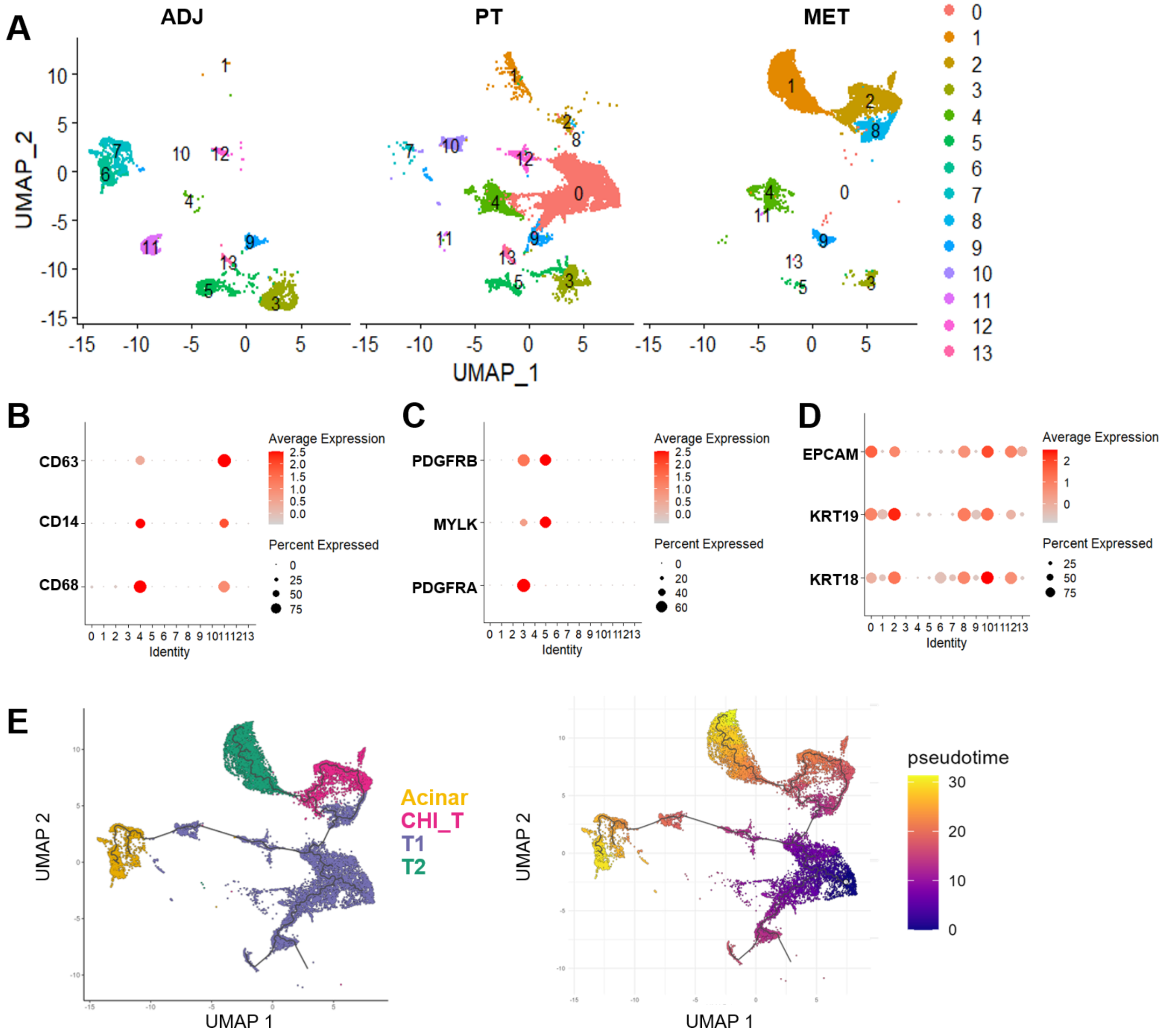

Figure S4. Single-cell transcriptomic profiling reveals cellular heterogeneity, gene expression signatures, and pseudotime-inferred transcriptional trajectories in human pancreatic tissues. (**A**) UMAP plot of the ADJ, PT, and MET samples composing 14 distinct cell populations. (**B-D**) A dot plot shows the relative expression of selected genes in the macrophages (B), fibroblasts (C), and epithelial cells (D) in human pancreatic tissue. (**E**) Pseudotime trajectory analysis of epithelial cell transitions projected on UMAP, colored by cell cluster identity (*Acinar*, *CHI_T*, *T1*, and *T2*; left) and pseudotime progression (right). With the trajectory root defined at T1. Solid lines indicate the inferred pseudotime ordering, which reflects transcriptional similarity rather than a confirmed lineage relationship.

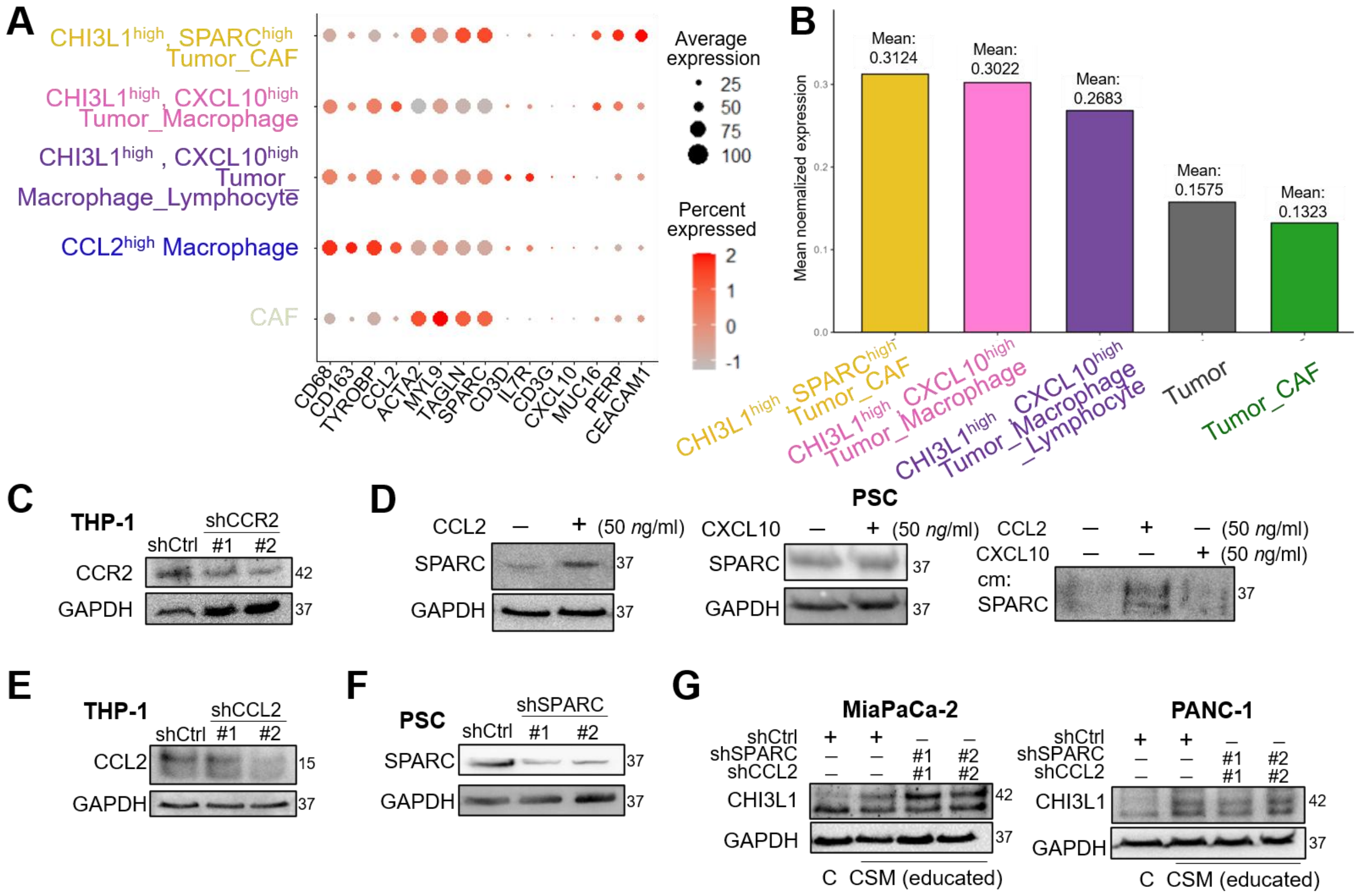

Figure S5. Spatial profiling and functional validation of CHI3L1-regulating signals in PDAC TME. (**A**) Dot plots depict the average expression levels of known marker genes across the indicated cell clusters. Dot size reflects the percentage of cells within each cluster that express the corresponding gene. (**B**) Mean normalized CHI3L1 expression across five tumor cell-containing spatial clusters (CHI3L1^{high}, SPARC^{high} Tumor_CAF, n = 71; CHI3L1^{high}, CXCL10^{high}, Tumor_Macrophage, n = 117; CHI3L1^{high}, CXCL10^{high} Tumor_Macrophage_Lymphocyte, n = 137; Tumor, n = 510; Tumor_CAF, n = 69), with percentage of CHI3L1-positive spots indicated above each bar. CHI3L1 expression differed significantly across clusters (Kruskal-Wallis, $P = 2.35 \times 10^{-6}$); pairwise Wilcoxon tests (BH-adjusted) confirmed that all three CHI3L1-high clusters had significantly higher expression than Tumor and Tumor_CAF (all $P < 0.05$), with no significant differences among the three CHI3L1-high clusters themselves (all $P > 0.44$). (**C**) Validation of shCCR2 (#1 and #2) in THP-1 by immunoblotting. (**D**) Intracellular (left and middle) and secreted (right) SPARC levels in PSCs treated with rCCL2 or rCXCL10 (50 *n*g/ml each). (**E, F**) Validation of shCCL2 (#1 and #2) (E) in THP-1 by immunoblotting, and validation of shSPARC (#1 and #2) (F) in PSC by immunoblotting. (**G**) CHI3L1 protein levels in MiaPaCa-2 and PANC-1 cells were assessed after culturing with shCtrl (C), CSM, or CSM collected from mixed culture conditions of MiaPaCa-2/PANC-1 with shCCL2 (#1 and #2) THP-1 and shSPARC (#1 and #2) PSC.

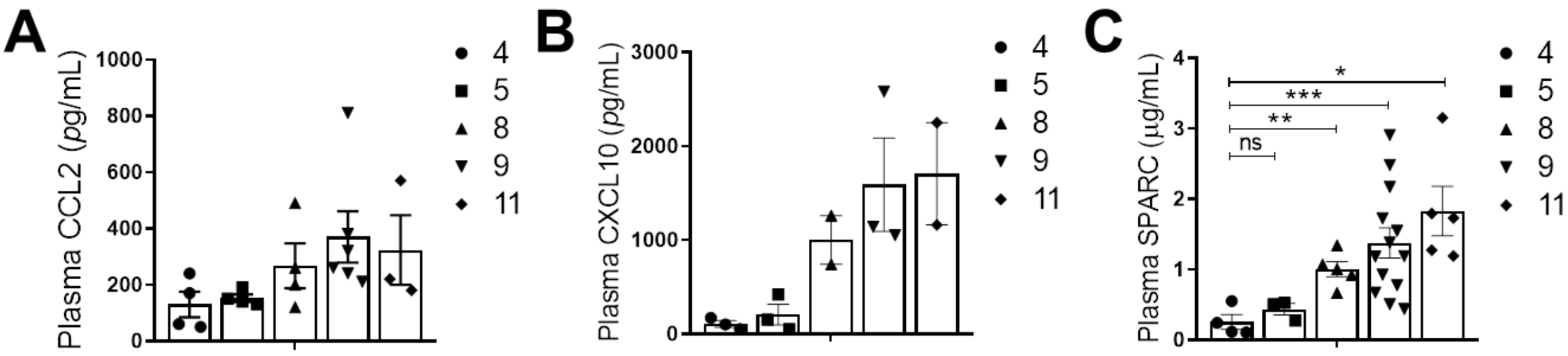

Figure S6. Circulating levels of CCL2, CXCL10, and SPARC during PDAC progression in KPPC mice. (**A**) ELISA quantification of plasma concentrations of CCL2 (n = 4, 4, 4, 6, 3 at weeks 4, 5, 8, 9, and 11, respectively); Welch's ANOVA $P = 0.24$; Dunnett's T3 vs. week 4, all $P > 0.05$. (**B**) Plasma CXCL10 (n = 3, 3, 2, 3, 2); presented descriptively without formal statistical testing due to limited sample size. (**C**) Plasma SPARC (n = 4, 3, 5, 13, 5); Welch's ANOVA $P = 0.001$; Dunnett's T3 vs. week 4: week 8, $P = 0.005$; week 9, $P = 0.001$; week 11, $P = 0.024$ (week 5, ns) in KPPC transgenic mice across disease progression (weeks 4 to 11). Data are shown as individual values with mean ± SD (* $P < 0.05$, ** $P < 0.01$, *** $P < 0.001$).

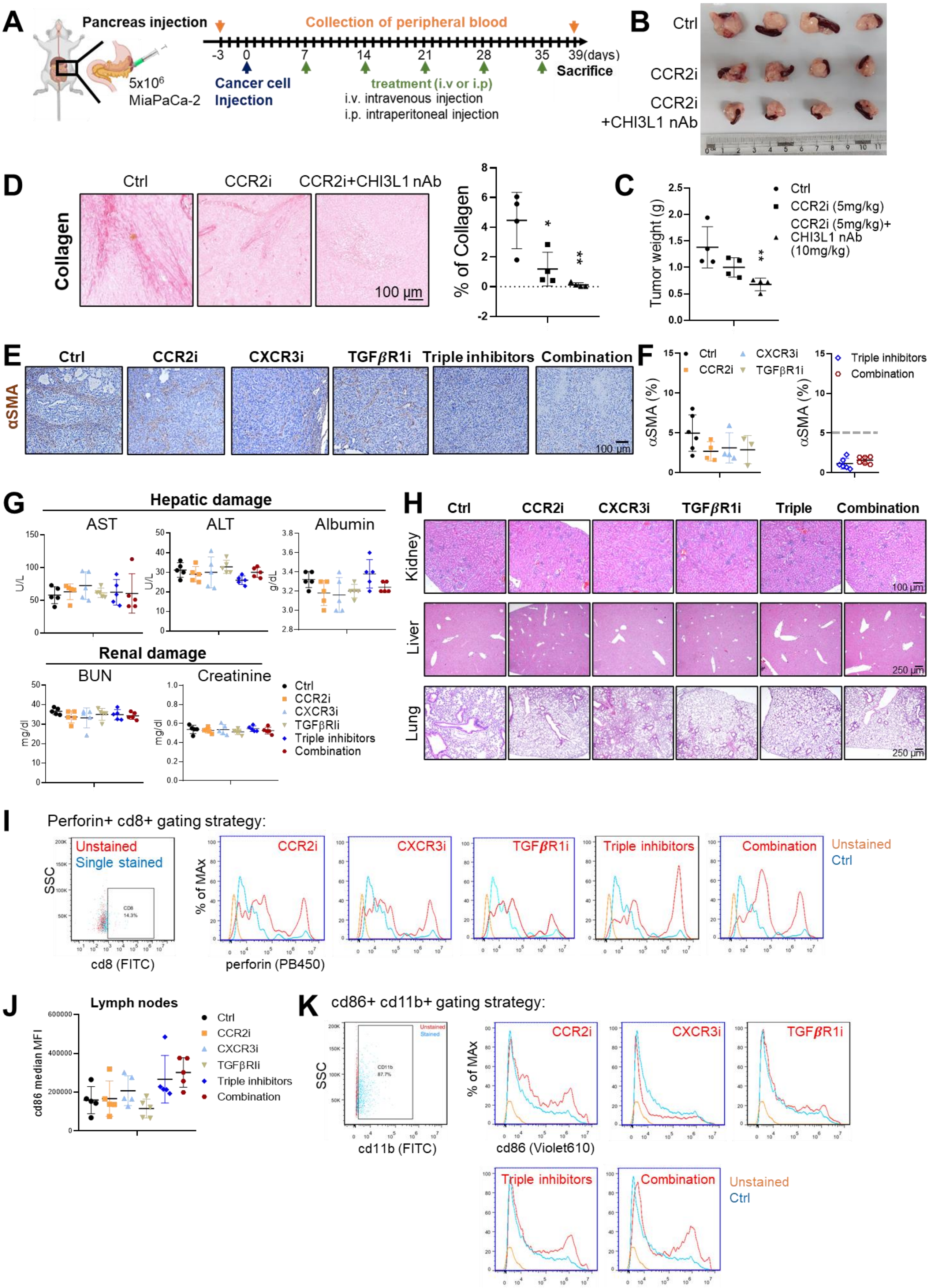
A
Pancreas injection
5x10^6 MiaPaCa-2
Collection of peripheral blood
-3 0 7 14 21 28 35 39(days)
Sacrifice
Cancer cell Injection
treatment (i.v or i.p)
i.v. intravenous injection
i.p. intraperitoneal injection
B
Ctrl
CCR2i
CCR2i +CHI3L1 nAb
C
Tumor weight (g)
Ctrl
CCR2i (5mg/kg)
CCR2i (5mg/kg)+ CHI3L1 nAb (10mg/kg)
D
Collagen
Ctrl
CCR2i
CCR2i+CHI3L1 nAb
100 μm
% of Collagen
E
αSMA
Ctrl
CCR2i
CXCR3i
TGFβR1i
Triple inhibitors
Combination
100 μm
F
αSMA (%)
Ctrl
CCR2i
CXCR3i
TGFβR1i
Triple inhibitors
Combination
G
Hepatic damage
AST
ALT
Albumin
U/L
g/dL
Renal damage
BUN
Creatinine
mg/dl
Ctrl
CCR2i
CXCR3i
TGFβRIi
Triple inhibitors
Combination
H
Ctrl
CCR2i
CXCR3i
TGFβR1i
Triple
Combination
Kidney
Liver
Lung
100 μm
250 μm
I
Perforin+ cd8+ gating strategy:
Unstained
Single stained
SSC
cd8 (FITC)
% of MAx
perforin (PB450)
CCR2i
CXCR3i
TGFβR1i
Triple inhibitors
Combination
Unstained
Ctrl
J
Lymph nodes
cd86 median MFI
Ctrl
CCR2i
CXCR3i
TGFβRIi
Triple inhibitors
Combination
K
cd86+ cd11b+ gating strategy:
SSC
cd11b (FITC)
% of MAx
cd86 (Violet610)
CCR2i
CXCR3i
TGFβR1i
Triple inhibitors
Combination
Unstained
Ctrl

Figure S7. Disrupting the CCR2/CXCR3/TGFβR1 axes together with CHI3L1 neutralization reduces PDAC tumor growth and shows no organ toxicity. (**A**) MiaPaCa-2 cells (5 x 10^6) were injected into the pancreas of NOD-SCID mice. Experimental design of cancer cell implantation, CCR2 inhibitor (CCR2i, 5 mg/kg), or CCR2i (5mg/kg) combined with CHI3L1 nAb (10mg/kg), collection of blood, and sacrifice. (**B**) Representative tumor images and (**C**) tumor weight. (**D**) PSR staining demonstrated the collagen levels across different treatment groups (Scale bar, 100 μm). (**E-F**) In the immunocompetent KPPC-luc model, representative αSMA IHC images (E) and quantification of αSMA^+ area (F) in the same tumors as in Fig. 7B, C (Scale bar, 100 μm); filled symbols represent individual animals with a tumor evaluable at sacrifice (Ctrl, n = 6; CCR2i, n = 4; CXCR3i, n = 4; TGFβR1i, n = 3), with group means ± SD and statistics; open symbols represent 6 ROIs from the single evaluable tumor in the triple-inhibitor and combination groups (technical replicates), plotted against the Ctrl mean (dashed line) for descriptive reference without formal statistical comparison. (**G**) Levels of plasma hepatic functional markers (AST, ALT, and albumin) and renal functional markers (BUN and creatinine) were measured on the day of sacrifice, with no significant differences observed between any treatment group and the Ctrl group. (**H**) H&E-stained sections of the kidneys, livers, and lungs were harvested on the day the mice were sacrificed. No obvious abnormalities were observed in the different treatment groups. (Scale bar, 100 or 250 μm). (**I**) Representative flow cytometry gating and overlay histograms of $perforin^+cd8^+$ T cells in lymph nodes across the indicated treatment groups corresponding to the quantification in Fig. 7E. (**J**) Flow cytometric quantification of cd86 median fluorescence intensity (MFI) in $cd11b^+$ myeloid cells in draining lymph nodes from mice receiving the indicated treatments. (**K**) Representative flow cytometry gating strategy and overlay histograms of $cd86^+cd11b^+$ myeloid cells in lymph nodes across the indicated treatment groups, corresponding to the quantification in (J). Data are presented as mean ± SD. *P* values were determined by one-way ANOVA (* $P < 0.05$, ** $P < 0.01$).

Table S1. The antibodies used in this study.

Target	KD	Raised in	Application	Dilution	Source	Catalog no.
CHI3L1	42	Rabbit	Western blot	1:1000	Cell signaling	#47066
	--[a]		IF-IHC	1:1000		
	--[a]				Abcam	ab259322
GAPDH	37	Mouse	Western blot	1:1000	Proteintech	60004-1-Ig
Actin	42	Mouse	Western blot	1:1000	GeneTex	GTX26276
Anti-rabbit IgG antibody (HRP)	--[a]	Goat	Western blot	1:4000	GeneTex	GTX213110-01
Anti-mouse IgG antibody (HRP)	--[a]	Goat	Western blot	1:5000	GeneTex	GTX213111-01
Phospho-NF-κB p65	65	Rabbit	Western blot/IF-ICC	1:1000	Cell Signaling	#3033S
	--[a]		ChIP	1:100		
Total- NF-κB p65	65	Rabbit	Western blot	1:1000	Cell Signaling	#8242S
Phospho-STAT3	75	Rabbit	Western blot	1:1000	Cell Signaling	#9145S

Total-STAT3	75	Mouse	Western blot	1:1000	Cell Signaling	#9139S
DNMT1	185	Rabbit	Western blot	1:1000	GeneTex	GTX116011
			ChIP	1:100		
CCL2	15	Rabbit	Western blot	1:1000	Elabscience	E-AB-68402
	-- [a]		IF-IHC	1:3000		
CXCL10	10	Rabbit	Western blot	1:1000	Abcam	ab306587
	-- [a]		IF-IHC	1:3000		
SPARC	37	Rabbit	Western blot	1:1000	Abclonal	A14494
	-- [a]		IF-IHC	1:5000		
CCR2	42	Rabbit	Western blot	1:1000	ELK Biotechnology	ES4365
CXCR3	40	Rabbit	Western blot	1:1000	ELK Biotechnology	ES2075
TGFβR1	56	Rabbit	Western blot	1:1000	ELK Biotechnology	ES3594
α-SMA	--[a]	Rabbit	DAB-IHC	1:500	GeneTex	GTX100034
	42		Western blot	1:1000		

CD206	--[a]	Rabbit	IF-IHC	1:1000	Abcam	ab64694
CD86	--[a]	Rabbit	IF-IHC	1:800	Abcam	ab243887
		Rat	Flow cytometry	1:200	BD Horizon	563055
Pan-cytokeratin	-- [a]	Rabbit	IF-IHC/ICC	1:1000	Abcam	ab86734
CK19	-- [a]	Mouse	IF-IHC	1:500	DAKO	M0888
CD68	-- [a]	Rabbit	IF-ICC	1:1000	Abcam	ab233172
CD8	-- [a]	Rabbit	IF-IHC	1:1000	Abcam	ab217344
		Rat	Flow cytometry	1:200	BD Horizon	553031
PD-1	-- [a]	Rabbit	IF-IHC	1:1000	GeneTex	GTX31309
LAG3	-- [a]	Rabbit	IF-IHC	1:1000	Abcam	ab237720
Granzyme B	-- [a]	Rabbit	IF-IHC	1:800	Cell Signaling	#46890T
DAPI	-- [a]	--[b]	IF-IHC/ICC	--[b]	GeneTex	GTX30920
CD11b	-- [a]	Rat	Flow cytometry	1:200	BD Horizon	564454

Perforin	-- [a]	Rat	Flow cytometry	1:200	BioLegend	154319

[a] Molecular weight is not applicable to this antibody in such an application.

[b] DAPI is a commercial product (Fluoroshield with DAPI) for nuclear staining.

Table S2. The primers used in this study.

Gene	Primer	Sequences (5’→3’)	Application[a]	PCR size (bp)	Tm (°C)
Human *CHI3L1* *mRNA*	Forward	CACACTCAAGAACAGGAACCCC	RT-qPCR	97	60
	Reverse	GGTGTTGGAGGCTATCTTGGAA			
Human *β-actin* *mRNA*	Forward	GGCGGCACCACCATGTACCCT	RT-qPCR	180	60
	Reverse	AGGGGCCGGACTCGTCATACT			
Human *CHI3L1* promoter NF-κB binding site 1	Forward	CAGAAGCAAAATAGCACCGG	ChIP	125	60
	Reverse	CTTTTGGGCCCGTGTCT			
Human *CHI3L1* promoter NF-κB binding site 2	Forward	CCTGTATTTCCTCCATGGTGC	ChIP[c]	114	60
	Reverse	CCAAGAATGCTTTAAGCCCC			
Human *CHI3L1* promoter NF-κB binding site 3	Forward	CCATTCTGCCTCTTGGAG	ChIP	123	60
	Reverse	CGTCCCTGTTTGTGGATAC			
Human *CHI3L1* promoter NF-κB binding site 4	Forward	GAGAGCTGCAAAACCAGC	ChIP[c]	104	60
	Reverse	CTCCAAGAGGCAGAATGGG			
Human *CHI3L1* promoter STAT3 binding site	Forward	CCTGGAGTGAATGCTGAAGATGC	ChIP	184	60
	Reverse	CTTCATGAGGCACCTGCCAG			
Human *CHI3L1* promoter containing no NF-κB binding site	Forward	TAGGTACCGTCTTTCCCTCTAAATAATCAGCATG	Construction	513	54
	Reverse	AGTGCTCGAGCAGAGGAGGGTTGAGAAAC			

Human *CHI3L1* promoter containing one NF-κB binding site	Forward	TAGGTACCCAGAAGCAAAATAGCACCGG	Construction	561	53
	Reverse	AGTGCTCGAGCAGAGGAGGGTTGAGAAAC			
Human *CHI3L1* promoter containing two NF-κB binding site	Forward	TAGGTACCCCTAGGAGTGATGAGAGATCAC	Construction	766	53
	Reverse	AGTGCTCGAGCAGAGGAGGGTTGAGAAAC			
Human *CHI3L1* promoter containing three NF-κB binding site	Forward	TAGGTACCCCATTCTGCCTCTTGGAG	Construction	929	54
	Reverse	AGTGCTCGAGCAGAGGAGGGTTGAGAAAC			
Human *CHI3L1* promoter containing four NF-κB binding site	Forward	TAGGTACCGAGCTGCAAAACCAGC	Construction	1013	54
	Reverse	AGTGCTCGAGCAGAGGAGGGTTGAGAAAC			
Human *CHI3L1* promoter NF-κB binding site 1 mutation	Forward	GGGAAGCTTCCTGTCTTTCCCTCTAAAT	site-directed mutagenesis	6217	49
	Reverse	GGAAGCTTCCCAAGAATGCTTTAAG			
Human *CHI3L1* promoter NF-κB binding site 2 mutation	Forward	TGGGATCCTCAAAACAGAAGCAAAATAGC	site-directed mutagenesis	6217	50
	Reverse	GAGGATCCCATAAAGAGAGAGGATC			
Human *CHI3L1* promoter NF-κB binding site 3 mutation	Forward	GATATCGTCACTCACTGCCTATTAATTAATG	site-directed mutagenesis	6217	50
	Reverse	GACGATATCAGAACTCCAAGAGG			

Human *CHI3L1* promoter NF-κB binding site 4 mutation	Forward	AAGAAGCTTTTGGACCCATTCTGCC	site-directed mutagenesis	6217	52
	Reverse	AAAGCTTTTCAAGAAAGTACCTTTATTTTTCAC			
Human *CHI3L1* promoter methylation	Forward	TTAGATGTTCGTGTAGTCGTTTCGT	MSP	241	49
	Reverse	CTAAACGCTCCCTTTTAAACCCGTA			
Human *CHI3L1* promoter unmethylation	Forward	TTAGATGTTTGTGTAGTTGTTTTGT	MSP	241	50
	Reverse	CTTGCAGTGATAGGTGATGTTCTGG			
Human *CHI3L1* bisulfite sequence 1	Forward	TTGTGAGATTTGAGGGTTAAGG	Pyrosequencing	289	56
	Reverse	CTCCCTATATTCCACTCATACTTATATATC			
	sequence	AGTTTTTTGTTTTTGATTTAGG		93	-- [b]
Human *CHI3L1* bisulfite sequence 2	Forward	AGAGTTGGGGAGTTGATTG	Pyrosequencing	338	56
	Reverse	CCCCTCACACTTTCTATCTTCAAAC			
	sequence	GGGGAGTTGATTGATG		93	-- [b]

[a] ChIP: chromatin-immunoprecipitation; MSP: methylation-specific polymerase chain reaction

[b] Tm is not applicable to this primer in such an application.

[c] ChIP amplicons targeting NF-κB sites 2 and 4 overlap substantially with the bisulfite sequence 1 region. Therefore, they are used to detect DNMT1 ChIP binding at the *CHI3L1* promoter.

Table S3. The plasmids and their characteristics used in this study.

Plasmid	Target	Insert (bp)	Function	Source
pEGFP-C1	None	–[a]	Vector control	Addgene
pEGFP-C1-NF-κb-p65	NF-κb-p65	1600	Overexpression	Addgene
pEGFP-C1-SP1	SP1	2358	Overexpression	Addgene
pCMV6-XL4	None	–[a]	Vector control	Origene
pCMV6-XL4-STAT3	STAT3	3384	Overexpression	Origene
pcDNA3-Flag	None	–[a]	Vector control	Dr. Ben-Kuen Chen
pcDNA3-Flag-c-Jun	c-Jun	1212	Overexpression	Dr. Ben-Kuen Chen
pcDNA3.1-V5	None	–[a]	Vector control	Dr. Li-Jung Juan
pcDNA3.1-hDNMT1-V5	DNMT1	4851	Overexpression	Dr. Li-Jung Juan

[a] The plasmid is used as a backbone vector and, therefore, has no inserted fragment.
